# The river blindness drug Ivermectin and related macrocyclic lactones inhibit WNT-TCF pathway responses in human cancer

**DOI:** 10.15252/emmm.201404084

**Published:** 2014-08-20

**Authors:** Alice Melotti, Christophe Mas, Monika Kuciak, Aiala Lorente-Trigos, Isabel Borges, Ariel Ruiz i Altaba

**Affiliations:** Department of Genetic Medicine and Development, University of GenevaGeneva, Switzerland

**Keywords:** cancer, Ivermectin, TCF, WNT, xenograft

## Abstract

Constitutive activation of canonical WNT-TCF signaling is implicated in multiple diseases, including intestine and lung cancers, but there are no WNT-TCF antagonists in clinical use. We have performed a repositioning screen for WNT-TCF response blockers aiming to recapitulate the genetic blockade afforded by dominant-negative TCF. We report that Ivermectin inhibits the expression of WNT-TCF targets, mimicking dnTCF, and that its low concentration effects are rescued by direct activation by TCF^VP^^16^. Ivermectin inhibits the proliferation and increases apoptosis of various human cancer types. It represses the levels of C-terminal β-CATENIN phosphoforms and of CYCLIN D1 in an okadaic acid-sensitive manner, indicating its action involves protein phosphatases.*In vivo*, Ivermectin selectively inhibits TCF-dependent, but not TCF-independent, xenograft growth without obvious side effects. Analysis of single semi-synthetic derivatives highlights Selamectin, urging its clinical testing and the exploration of the macrocyclic lactone chemical space. Given that Ivermectin is a safe anti-parasitic agent used by > 200 million people against river blindness, our results suggest its additional use as a therapeutic WNT-TCF pathway response blocker to treat WNT-TCF-dependent diseases including multiple cancers.

## Introduction

The identification of cell–cell communication signaling pathways that regulate embryogenesis and adult tissue homeostasis, and which are altered in hyperproliferative or degenerative diseases, has lead to the idea that specific pathway modulators may be efficacious therapeutic agents. For example, a variety of sporadic human cancers have been found to harbor hyperactive canonical WNT signaling, leading to the constitutive activation of β-CATENIN, a key polyvalent protein that is modified by phosphorylation at multiple sites. In particular, early colon cancers commonly display loss of function of the tumor suppressor Adenomatous polyposis coli (APC), a key component of the β-CATENIN destruction complex, or harbor N-terminal gain of function mutations in β-CATENIN that render it APC-resistant. In both of these cases, this leads to canonical WNT pathway activation and the resulting regulation of target genes (reviewed in Kinzler & Vogelstein, 1996; MacDonald*et al*, 2009; Valenta*et al*, 2012; Varnat*et al*, 2010). Other cancers also show an active canonical WNT pathway; these include carcinomas of the lung, stomach, cervix, endometrium, and lung as well as melanomas and gliomas (e.g., Nguyen*et al*, 2009; Kandoth*et al*, 2013; http://cancer.sanger.ac.uk/cancergenome/projects/cosmic/).

In normal embryogenesis and homeostasis, the canonical WNT pathway is activated by secreted WNT ligands produced in highly controlled context-dependent manners and in precise amounts. WNT activity is transduced in the cytoplasm, inactivates the APC destruction complex, and results in the translocation of activate β-CATENIN to the nucleus, where it cooperates with DNA-binding TCF/LEF factors to regulate WNT-TCF targets and the ensuing genomic response (e.g., Kinzler & Vogelstein, 1996; Shitashige*et al*, 2008; MacDonald*et al*, 2009; Clevers & Nusse, 2012; Valenta*et al*, 2012).

In the absence of WNT ligands, the APC destruction complex acts to phosphorylate cytoplasmic β-CATENIN in its N-terminal region (including at Ser33/Ser37 via CK1 and GSK3 β kinases) targeting it for proteasomal degradation. Without active nuclear β-CATENIN, there is no activation of positive WNT-TCF targets. In contrast, under active WNT signaling, β-CATENIN escapes cytoplasmic degradation and is phosphorylated at its C-terminus, for instance at positions Ser552 and Ser675, by various kinases including AKT, PAK, PKA, and possibly AMPK. Such C-terminal phosphorylation has been found to be essential for the transcriptional function of β-CATENIN with TCF factors (Hino*et al*, 2005; Taurin*et al*, 2006; Fang*et al*, 2007; Zhao*et al*, 2010; Zhu*et al*, 2012a). These findings indicate that beyond the loss of activity of the APC destruction complex, for instance through*APC* mutation, phosphorylation of β-CATENIN at C-terminal sites is required for the full activation of WNT-TCF signaling and the ensuing WNT-TCF responses in cancer.

Attempts to pharmacologically inhibit WNT signaling have lead to the identification of a number of small molecules that act at different levels in the core signaling cascade (e.g., Lepourcelet*et al*, 2004; Curtin & Lorenzi, 2010; Anastas & Moon, 2013; see http://www.stanford.edu/group/nusselab/cgi-bin/wnt/smallmolecules). These include inhibitors of Porcupine and Tankyrases, which promote normal WNT ligand secretion or cytoplasmic transduction, respectively (Huang*et al*, 2009; Dodge*et al*, 2012; Waaler*et al*, 2012; Lau*et al*, 2013). However, activation of WNT signaling below the level of ligand function or cytoplasmic transduction, as in the case of loss of APC for instance (see above), would appear to complicate the effects of pathway blockade at upstream levels. So far, there are no approved WNT signaling inhibitors in the clinics and clinical trials of new lead compounds are lengthy, very costly, and with less than 1/20 success rates.

Here, we have thus focused on the end part of the WNT-TCF pathway, using a well-established transcriptional reporter assay (Barker & Clevers, 2006) for TCF activity driven by activated, APC-insensitive N-terminal mutant β-CATENIN (N'Δ45 β-CATENIN). However, with the aim to find blockers of WNT-TCF responses in cancer, we implemented two key modifications: i- a reliable dynamic range determined by activated β-CATENIN as the activating pole and activated β-CATENIN plus dominant-negative TCF (dnTCF) as the repressing pole, and ii- sine qua non mimicry of genetic blockade by dnTCF activity for any hit.

We find that macrocyclic lactones of the Avermectin family have specific anti-WNT-TCF response activity in human cancer cells and that the clinically approved compound Ivermectin (EMEA- and FDA-approved) is a specific WNT-TCF response blocker at low micromolar concentrations.

Ivermectin and other macrocyclic lactones are well-tolerated agents that have been used to treat millions of people for river blindness (Thylefors, 2008; Traore*et al*, 2012) and other parasite infections (Campbell*et al*, 1983; González*et al*, 2012; Nolan & Lok, 2012). These actions of Ivermectin have been ascribed to its blockade of parasite ligand-gated chloride channels (Hibbs & Gouaux, 2011; Lynagh & Lynch, 2012). The WNT-TCF response blockade that we describe for low doses of Ivermectin suggests an action independent to the deregulation of chloride channels. Its low concentrations effects are rescued epistatically by direct constitutive activation of TCF transcriptional activity and involve the repression of the levels of C-terminally phosphorylated β-CATENIN forms and of CYCLIN D1, a critical target that is an oncogene and positive cell cycle regulator. Moreover, the Avermectin single-molecule derivative Selamectin, a drug widely used in veterinarian medicine (Nolan & Lok, 2012), is ten times more potent acting in the nanomolar range. Finally, Ivermectin has*in vivo* efficacy against human colon cancer xenografts sensitive to TCF inhibition with no discernable side effects. The ability of systemic Ivermectin to also block lung cancer growth*in vivo* supports the possible use of Ivermectin in particular, and other macrocyclic lactones such as Selamectin in general, as WNT-TCF pathway response blockers to combat WNT-TCF-dependent human diseases including cancers of the intestine, breast, skin, and lung.

## Results

### Identification of drugs that block WNT-TCF pathway responses in human cancer cells mimicking the effects of dnTCF

**Figure 1:**
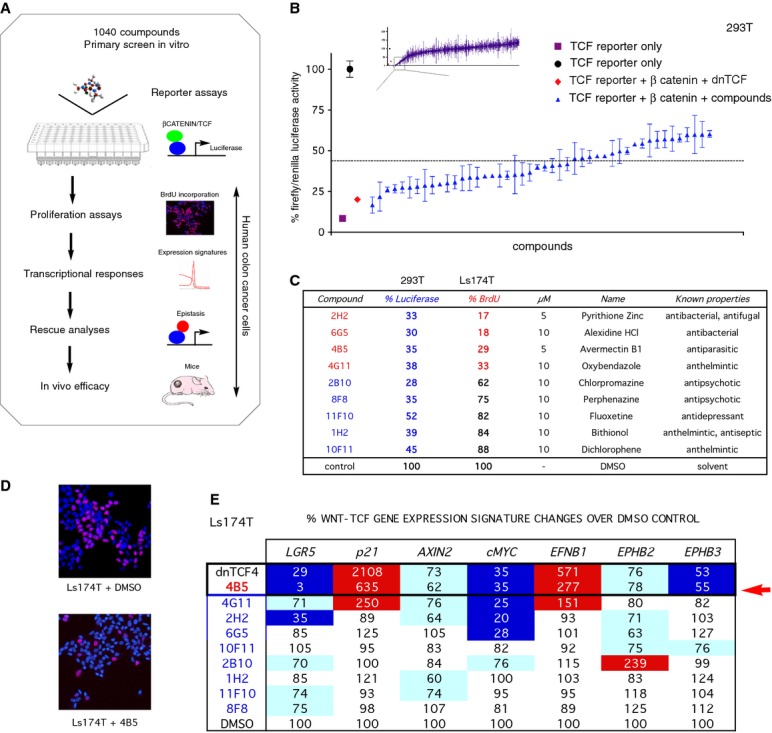
Identification of small molecule β-CATENIN-TCF antagonists Flow chart outlining the sequential assays leading to the selection of small molecules with anti-β-CATENIN/TCF and anti-tumor activity. Small molecules from a 1040 USP approved library were first selected based on their activity in a medium–high-throughput luminescent assay in human 293T cells using N-terminally truncated N'Δ45β-CATENIN and a multimerized TCF-binding site → luciferase reporter. Positive compounds were then submitted to a series of selection steps using human cancer cells comprising from top to bottom: (i) The inhibition of cancer cell proliferation in BrdU incorporation assays; (ii) The inhibition of a TCF target gene signature as determined by qRT-PCR in cancer cell lines and in primary cancer cells; (iii) The epistatic rescue by dominant-active TCF activity; (iv) The*in vivo* efficiency of hits evaluated via IP injections into NMRI Nude mice carrying subcutaneous cancer xenografts.Quantitative chart of firefly/Renilla luciferase levels in TCF reporter assays in 293T cells resulting from testing the library compounds. The upper inset shows the distribution of hits from the library and the lower graph a magnification of 27 hits below 45% (representing 2.5% of the total number of compounds), as compared with the level of TCF reporter plus N'Δβ-CATENIN with DMSO control (black dot) that is equated to 100%. Additional controls present in each plate were as follows: TCF reporter alone as negative control (purple square), and TCF reporter plus N'Δβ-CATENIN plus dnTCF as positive control (red diamond). 1,040 compounds were first screened individually at 10 μM in duplicate in independent plates and the results scored and represented by their mean (blue triangles) with individual values (as top and bottom bars) noted. Toxic compounds that fully scored below the level of dnTCF activity (red diamond) are not shown. In all assays, firefly luciferase activity was normalized by Renilla luciferase reporter values, Renilla luciferase was driven by the ubiquitous viral thymidine kinase promoter.Table of the top 9 antagonist candidates with their code number in the MicroSource 1040 list, micromolar concentration used, name, general known properties and inhibition of TCF-luciferase reporter activity and BrdU incorporation in 293T and colon cancer Ls174T cells, respectively. Both values are shown as percent reduction over DMSO controls shown at the bottom (equated to 100%). Note that only 4 (red) of the selected 9 hits decrease BrdU incorporation and thus the proliferation of human colon cancer cells by more than 50%.Micrographs of immunochemical labeling of Ls174T cells after anti-BrdU antibody (red) labeling and DAPI (blue) staining to show the extent of cell replication (BrdU incorporation, red) and total cell numbers (with nuclei stained blue). Results are shown for treatment with DMSO as control (top) and 4B5 (bottom) at 10 μM for 48 h. Scale bar = 50 μM.Heat map of mRNA expression values in human colon cancer Ls174T cells as determined by RT-qPCR for a 7-gene WNT-TCF signature. All values are percentages of experimental over control (DMSO-treated only) ratios after normalization of individual gene expression Ct values over those of housekeeping genes. As positive control, as our genetic benchmark, the expression levels driven by dnTCF expression 24 h after transfection are shown. Samples treated with drugs were collected 12 h after treatment. Expression changes are highlighted as follows: Dark blue: repression at or below 55%. Light blue: repression below 80%. Red: enhancement above 150%. Only 4B5 (red arrow) tracks the complete signature changes produced by dnTCF.

We have used a transcriptional reporter assay for TCF activity driven by APC-insensitive N'Δβ-CATENIN, to test a collection of clinical-trial tested small molecules (Microsource 1040 library) (Fig1A). The optimized assay included a multimerized TCF-binding site firefly luciferase reporter in human 293T cells with a fivefold dynamic range as determined by maximal stimulation with active N'Δβ-CATENIN and maximal repression with N'Δβ-CATENIN plus dnTCF (see Varnat*et al*, 2010) (Fig1A). All assays also included a Renilla luciferase control for normalization (see Materials and Methods). Mutant TCF-binding site reporter controls for non-specific effects on general transcription were not part of the screen in order to streamline it, but this concern was directly addressed by testing the endogenous transcription of housekeeping genes by rt-qPCR (see below). The primary screen yielded a hit rate of 2.6% with > 55% inhibitory activity (Fig1B). This excluded 3.3% of compounds considered cell-toxic as they repressed luciferase activity below that afforded by dnTCF.

The top nine candidates in the screen (Fig1B, Supplementary Fig S1)—showing the highest repressed levels of normalized TCF-luficerase reporter activity above the baseline given by dnTCF—were then selected and tested for inhibition of human Ls174T colon cancer cell proliferation, as this is WNT-TCF-dependent (van de Wetering*et al*, 2002). BrdU incorporation analyses showed that only four out of the selected nine compounds repressed cell proliferation at > 50% as compared with DMSO-treated control cells (Fig1C and D), yielding a secondary hit rate of ˜ 0.4%.

Tertiary analyses measured the mRNA levels of an established cohort of canonical WNT-TCF target genes in Ls174T cells (van de Wetering*et al*, 2002; Hatzis*et al*, 2008) after 12 h of treatment to highlight early responses. Blockade of WNT-TCF by dnTCF4, used throughout our study as the genetic benchmark, yielded a pattern of gene expression that included both downregulated and upregulated genes, in comparison with control cells (Fig1E). Expression levels were normalized by those of the ‘housekeeping’ genes*TBP* and*HMBS* (see Materials and Methods). The expression of these genes was unaffected by the candidate compounds, providing an additional control against general transcriptional effects.

Of the 4 putative antagonists, only one, 4B5 (Avermectin B1), perfectly tracked the gene expression profile induced by dnTCF4 (Fig1E, arrow). 4G11 (Oxybendazole) yielded a similar signature but failed to repress*LGR5* to the same extend as 4B5 (Fig1E). Although it harbors partial WNT-TCF antagonist activity, commercial Oxybendazole was largely inactive (B. Petcova, CM and ARA, data not shown). As in Ls174T cells, 4B5 treatment modified TCF targets and cell proliferation in primary human local colon cancer TNM4 CC14 cells (without detected mutations in*APC* or*β-CATENIN*) and in primary colon cancer liver metastasis mCC11 cells (Varnat*et al*, 2009) in a dose-dependent manner (Supplementary Fig S2). The final hit rate of the screen was ˜ 0.1%.

### Anti-proliferative activity of Abamectin, Ivermectin, Selamectin, and related macrocyclic lactones on human cancer cells

**Figure 2:**
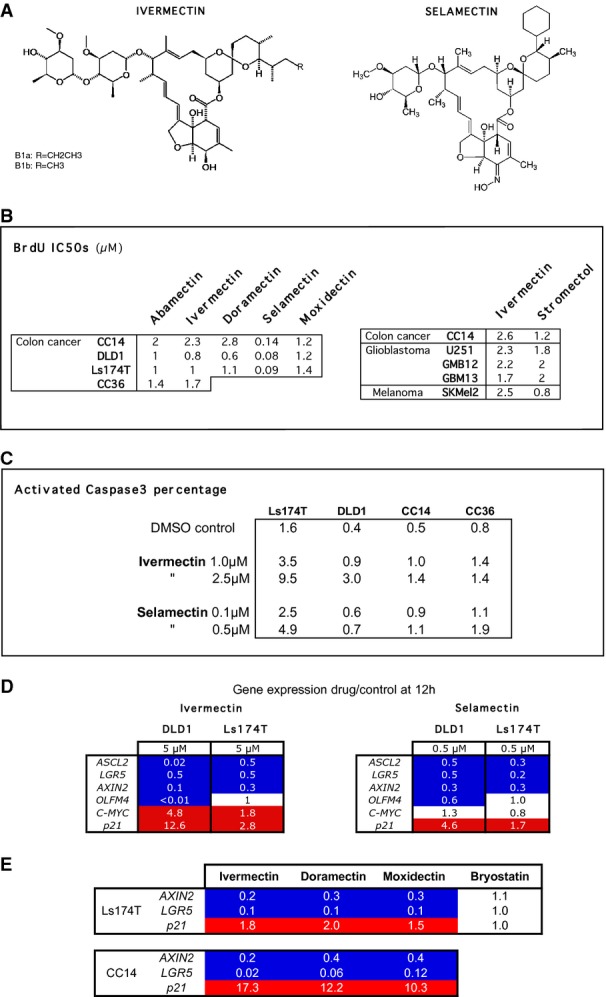
Anti-proliferative and pro-apoptotic effects of Ivermectin, Selamectin, and related macrocyclic lactones, and repression of WNT-TCF targets Chemical structures of Ivermectin and Selamectin. Note that ivermectin is a fermentation mixture with the two major forms denoted by R.Tables of BrdU incorporation IC_50_s, calculated with PRISM from triplicated data shown in Supplementary Fig S3. Stromectol™ is a commercial name of Ivermectin from the local pharmacy. Results are shown for multiple human cancer types and multiple human colon cancer cells as noted.Table of the percentage of activated Caspase3^+^ cells in different cell types after treatment with different drug concentrations as noted.Heat maps of mRNA expression levels of WNT-TCF targets shown as ratios of housekeeping gene-normalized Ct values from cells under different treatments over normalized Ct values of DMSO-treated control DLD1 or Ls174T cells. No change over control is shown as a value of 1. Dark blue: repression of expression at or below 0.6. Red: enhanced expression above 1.5-fold.Heat map as in (D) showing the changes in the expression for three TCF targets in two colon cancer cell types after treatment with 5 μM Ivermectin, Doramectin, Moxidectin, or Bryostatin for 12 h, compared with DMSO control sibling cells taken at the same time.

4B5 represents the anti-helmintic agent Avermectin B1, which belongs to the 16-membered Avermectin macrocyclic lactone family derived from*Streptomyces avermitilis*. It is a fermentation mixture of > 80% Avermectin B1a (5-O-demethyl-Avermectin A) and < 20% B1b (5-O-demethyl-25-de(1-methylpropyl)-25-(1-methylethyl-Avermectin A1) (S3). Ivermectin is a clinically approved Avermectin B1 derivative (> 90% 22,23-dihydroavermectinB1a and < 10% of 22,23-dihydroavermectinB1b) (Fig2A, Supplementary Fig S3), used in humans against insect and worm infections, including river blindness caused by*Onchocerca volvulus* (Thylefors, 2008; Traore*et al*, 2012).

Ivermectin showed similar IC_50_s on BrdU incorporation*in vitro* across several human colon cancer cells (IC_50_: 1–2.4 μM) as Abamectin—the commercial name of Avermectin B1 (IC_50_: 0.8–2 μM) (Fig2B, Supplementary Fig S3). Likewise, Ivermectin and its commercial form from the pharmacy, Stromectol™, showed similar inhibition of BrdU incorporation*in vitro* in two primary human glioblastomas and two human melanoma cell lines as compared with colon cancer cells (Fig2B, Supplementary Fig S3).

Given that Avermectin B1 and Ivermectin are mixtures, we tested two commercial single-molecule Avermectin A1a derivatives, Doramectin, and Selamectin, to explore the activity of pure macrocyclic compounds. Doramectin (25-cyclohexyl-5-O-demethyl-25-de(1-methylpropyl) Avermectin A1a) and Selamectin (25-cyclohexyl- 4′-O-de(2,6-dideoxy-3-O-methyl-α-L-arabino-hexopyranosyl)- 5-demethoxy-25-de(1-methylpropyl)- 22,23-dihydro-5-(hydroxyimino)- Avermectin A1a) (Fig2A and B, Supplementary Fig S3) are widely used in veterinarian medicine as anti-parasitics (Nolan & Lok, 2012). Doramectin was as effective as Ivermectin on reducing the proliferation of various human colon cancer cells (IC_50_: 0.6–2.8 μM; Fig2B, Supplementary Fig S3). In contrast, Selamectin, which scored as toxic in the primary screen at 10 μM, was ˜ tenfold more potent, showing nanomolar BrdU incorporation IC_50_s (0.08–0.14 μM, Fig2B, Supplementary Fig S3). Tested in parallel, the Milbemycin macrocyclic lactone family member Moxidectin also displayed comparable anti-proliferative activity to Ivermectin (Fig2B, Supplementary Fig S3). However, not all macrocyclic lactones showed such activity as Bryostatin, a distant macrocyclic lactone, was largely ineffective (90 and 88% BrdU incorporation vs. controls in DLD1 cells at 2.5 and 5 μM, respectively).

### Ivermectin and Selamectin induce apoptosis

Ivermectin and Selamectin (Fig2A) were chosen for further study given the EMEA- and FDA-approved status of the first and the high potency*in vitro* and widespread veterinarian use of the second. Activated Caspase3 was used as a marker of apoptosis by immunohistochemistry 48 h after drug treatment. Selamectin and Ivermectin induced up to a sevenfold increase in the number of activated Caspase3^+^ cells in two primary (CC14 and CC36) and two cell line (DLD1 and Ls174T) colon cancer cell types (Fig2C). All changes were significative (*P* < 0.05) with the exception of 0.1 μM Selamectin in DLD1, CC14 and CC36, and were concentration and cell-type-dependent, with strongest effects detected in DLD1 cells with 2.5 μM Ivermectin (Fig2C).

### Ivermectin and Selamectin repress the expression of positive direct WNT-TCF targets

Gene expression analyses were performed 12 h after initiation of treatment to detect early responses. Importantly, both treatments repressed the direct positive WNT-TCF targets*AXIN2*,*LGR5*, and*ASCL2* in DLD1 and Ls174T colon cancer cells, although they had variable effects on*cMYC* (Fig2D). Minor differences in the levels of individual components of a WNT-TCF signature in different cells are expected (Herbst*et al*, 2014). In addition, both treatments also enhanced the levels of*p21,* a cell cycle blocker. This plus the finding that the expression of the ‘housekeeping’ genes*TBP* and*HMBS* used for normalization was unaffected, indicated the absence of general toxic effects on transcription.

### Comparison of the effects of Ivermectin, Doramectin, Moxidectin, and Bryostatin on TCF targets in colon cancer cells

The inhibition of proliferation observed after treatment with different macrocyclic lactones (see above) begged the question of their relative abilities to block TCF responses in colon cancer cells. Analysis of the TCF targets*AXIN2, LGR5*, and*p21* revealed equal potency of Ivermectin, Doramectin, and Moxidectin in Ls174T cells (Fig2E). The distantly related macrocyclic lactone Bryostatin was inactive (Fig2E). In primary CC14 cells, Ivermectin gave the strongest repression of*AXIN2* and*LGR5* and the strongest activation of*p21*, followed by Doramectin and by Moxidectin, all at the same concentration (Fig2E). TCF target analyses (Fig 2D and E) thus confirmed the choice of Ivermectin and Selamectin for further studies.

### Ivermectin and Selamectin inhibit clonogenic self-renewal of colon cancer stem cells

**Figure 3:**
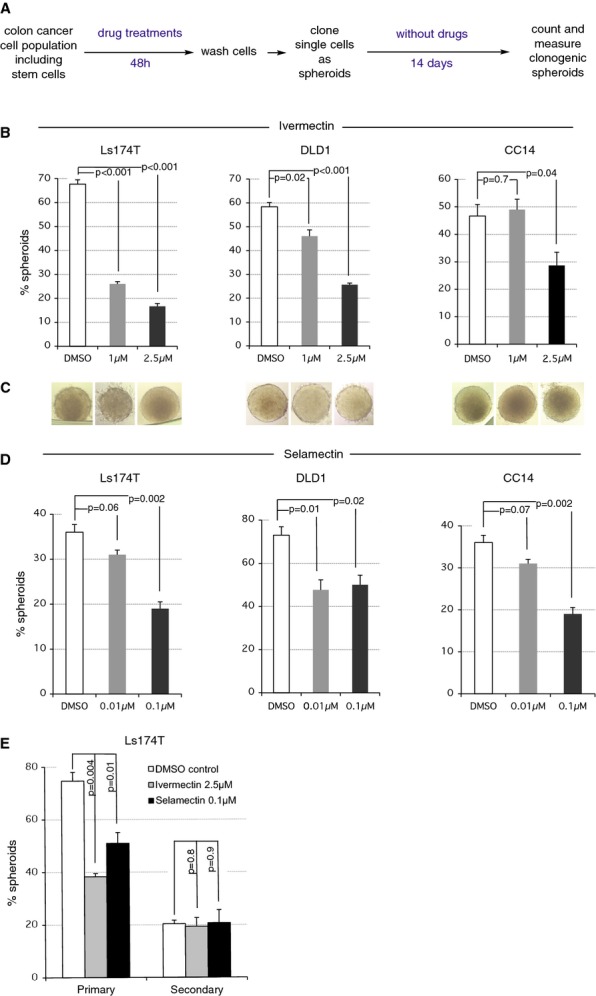
Pre-treatment with Ivermectin and Selamectin inhibits colon cancer stem cell self-renewal in clonogenic spheroid assays A Work flow for the pre-treatment and assessment of colon cancer stem cell self-renewal by*in vitro* clonogenic assays. Note that in this set-up, cells are treated before they are challenged to make floating clonogenic spheroids of human colon cancer cells (colon spheroids). B–D (B,D) Histograms of the number of colon spheroids per 96-well plate, of DLD1 and Ls174T human colon cancer cell lines as well as of primary CC14 human colon cancer cells, after treatments with DMSO or different concentrations of Ivermectin (B) or Selamectin (D) as noted, following the scheme shown in (A). Columns show averages of triplicate experiments, with 3 plates per experiment. DMSO-treated cells are shown as controls. (C) Representative images of colon spheroids after 14 days (see A). Each panel corresponds to the treatment immediately above the image. Scale bar = 100 μM. E Histograms of the number of Ls174T colon cancer cell spheroids obtained under the noted conditions from 2D drug-pretreated cells (primary cloning) and after a second clonogenic assay using as starting material the primary cloning spheroids. Data information: Error bars = s.e.m. Probability (*P*) values are derived from two-tailed*t*-tests.

The strong downregulation of the expression of the intestinal stem cell genes*ASCL2* and*LGR5* (van der Flier*et al*, 2009; Schepers*et al*, 2012; Zhu*et al*, 2012b) by Ivermectin and Selamectin (Fig2D) raised the possibility that these drugs could affect WNT-TCF-dependent colon cancer stem cell behavior. We thus tested for the ability of these drugs to antagonize cancer stem cell-driven clonogenic spheroid formation*in vitro*, which measures the ability of a cancer stem cell to self-renew and give rise to other progeny. Pretreatment of DLD1, CC14, and Ls174T attached cells in 2D culture with Ivermectin (1–2.5 μM) or Selamectin (0.01–0.1 μM) (Fig3A) diminished the frequency of subsequent clonal floating spheroids by up to 73 and 43%, respectively, as compared with control, without affecting spheroid size (Fig3B–E). Whereas this assay tests for an inhibitory effect of Ivermectin on the self-renewal of cancer stem cells in 2D culture that can then give rise to 3D spheroids, it does not directly address spheroid growth, that is self-renewal of the founding cancer stem cells or proliferation of derived non-stem cells in 3D culture.

To investigate the possibility that Ivermectin also affects proliferation in 3D spheroids as it does in 2D cultures, the drug was added at the beginning of clonal spheroid growth so that it would be present during spheroid formation. In this second test, Ivermectin decreased spheroid frequency in a dose-dependent manner (Supplementary Fig S4). Taken the results of the two distinct assays together, the data indicate that Ivermectin and Selamectin affect the frequency of clonogenic events*in vitro* as well as the growth of resulting clonal spheroids. These results suggest an action on both the bulk of the tumor and its cancer stem cells. Indeed, analyses of primary vs. secondary clonogenic events in Ls174T revealed that Ivermectin pre-treatment only affected primary cloning, without effects on secondary events far removed from the time of drug action (Fig3F). This result also indicates the absence of long-lasting adverse drug effects.

Colon cancer cells with clonogenic spheroid capacity often express high levels of the CD133 (the AC133) epitope as compared with non-clonogenic cells (e.g., Varnat*et al*, 2009). Quantification of CC14 CD133^+^ cells by magnetic activated cell sorting after dissociation of 2D cultures with limited Trypsin or StemProAccutase treatments revealed no differences between control vs. (2.5 and 5 μM) Ivermectin-treated cells (7% vs 6.3%;*P* > 0.05). Ivermectin treatment thus separates CD133 expression and spheroid clonogenicity.

### Ivermectin selectively represses TCF-dependent human colon cancer xenograft growth*in vivo*

Both genetic models of cancer and human xenografts provide highly valuable tools to model human disease (Richmond & Su, 2008). However, since we sought to test the effects of Ivermectin on human tumor cells, we chose to perform xenograft experiments.

There is general requirement of WNT-TCF signaling for human colon cancer cell proliferation*in vitro* (e.g., van de Wetering*et al*, 2002; Varnat*et al*, 2010), but this is not the case*in vivo*. We have shown that blocked WNT-TCF activity by expression of dnTCF4 in human colon cancer cells and primary xenografts in mice (including tumors generated by grafted Ls174T cells and primary CC14, CC36 cells) does not generally lead to arrested growth or cell death, with the exception of DLD1 cells (Varnat*et al*, 2010). We have thus used DLD1 vs. CC14 subcutaneous xenografts in Nude mice as a stringent differential test for the activity and specificity of Ivermectin*in vivo*. We reasoned that if this drug phenocopies genetic blockade of WNT-TCF responses by dnTCF in human epithelial cancer cells, it should block the growth of*in-vivo*-dnTCF-sensitive DLD1 tumors but not that of*in-vivo*-dnTCF-insensitive CC14 xenografts.

**Figure 4:**
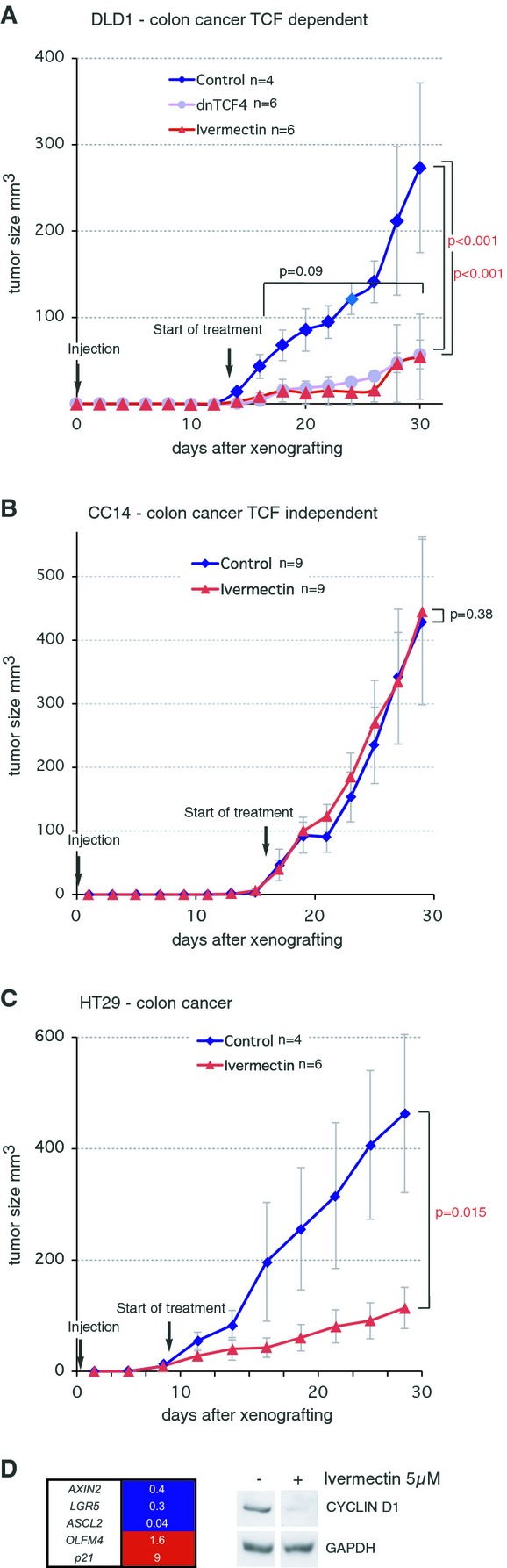
Ivermectin specifically blocks TCF-dependent tumor growth*in vivo* Tumor growth curves plotting tumor size over days. Subcutaneous xenografts in Nude mice of human colon cancer (DLD1, CC14, HT29) cells were treated by IP injection of cyclodextrin-conjugated Ivermectin or cyclodextrin carrier alone after the visual detection of tumors (9–19 days after injection depending on the cell type). Xenografted mice were randomly separated into experimental and control cohorts and treated. Arrows denote the start of treatments. Mice were injected daily IP at 10 mg/kg. Inhibitory effects of Ivermectin and its mimicry of the effects of dnTCF on*in-vivo*-TCF-dependent DLD1 xenografts.Lack of activity of Ivermectin on*in-vivo*-TCF-independent CC14 xenografts (see Varnat*et al*, 2010).Inhibitory action of Ivermectin on colon cancer HT29 xenografts.Heat map of gene expression shown as ratios of experimental over controls after housekeeping gene normalization, determined by RT-qPCR, revealing the repression of WNT-TCF targets by Ivermectin in HT29 cells. Right panel: Western blots showing the repression of CYCLIN D1 protein levels by Ivermectin treatment of HT29 cells. Control GAPDH levels are shown for each condition. CYCLIND1 and GAPDH panels come from the same Western blots. Data information: Error bars = s.e.m.*n* = number of tumors per condition.*P*-values are from*t*-tests.

Intraperitoneal injections of cyclodextrin-conjugated Ivermectin given daily at 10 mg/kg after tumor establishment inhibited DLD1 tumor growth as compared with cyclodextrin carrier-only injected mice xenografted with sibling cells (Fig4A). Critically, tumor inhibition by Ivermectin was equal to that observed with dnTCF4, our genetic benchmark (Fig4A). Moreover, Ivermectin did not affect the growth of*in-vivo*-dnTCF4-insensitive CC14 tumors (Fig4B). Injected mice showed no side effects and treatment with Ivermectin. Treatment with the commercial clinical-grade form of Ivermectin, Stromectol™, also had positive effects (Supplementary Fig S5). The inhibition of DLD1 tumors, however, was size-dependent as starting the treatment when subcutaneous xenografts were large (> 100 mm^3^) yielded a poor response (8/9) to Ivermectin treatment via IP (not shown), possibly due to ineffective drug penetration.

To extend*in vivo* data to a second WNT-dependent colon cancer type, we used HT29, which harbors a similar truncation in APC as DLD1 cells. Subcutaneous injection of HT29 cells into the flanks of Nude mice yielded tumors, the growth of which was repressed by Ivermectin treatment (Fig4C). Moreover, the overall pattern of TCF target gene regulation in HT29 cells in response to Ivermectin treatment was overall similar to that detected in DLD1 cells (Fig2D and 4D).

### Ivermectin inhibits lung cancer xenograft growth*in vivo*

Beyond colon cancer, WNT-TCF signaling has been implicated in a number of other tumor types including advanced non-small cell lung cancer (Nguyen*et al*, 2009; Pacheco-Pinedo*et al*, 2011). We have therefore used H358 human metastatic lung bronchioalveolar carcinoma cells to test for a response to Ivermectin at identical doses as for colon cancer cells. Pre-established H358 tumors responded to Ivermectin showing a ˜ 50% repression of growth (Supplementary Fig S5). Consistently, Ivermectin treatment repressed a lung cancer WNT-TCF signature that included the direct targets*AXIN2*,*LEF1, SOX4*, and*CYCLIND1* (as*ASCL2* and*LGR5* are not expressed in these cells) and enhanced*p21* levels (Supplementary Fig S5). Ivermectin also diminished the protein levels of CYCLIN D1, a direct TCF target and oncogene, in both HT29 and H358 tumor cells (Fig4D, Supplementary Fig S5).

### Direct activation of TCF rescues the effects of low doses of Ivermectin and Selamectin

**Figure 5:**
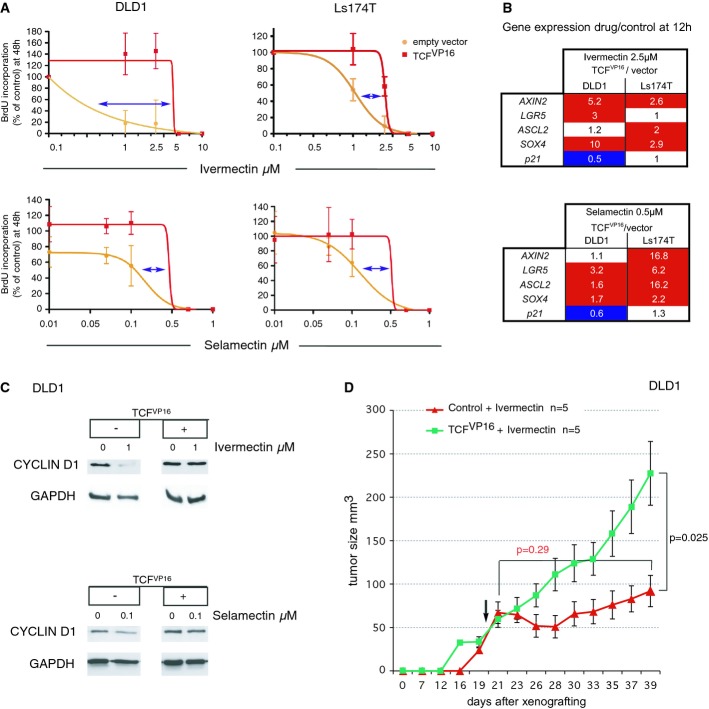
Epistatic rescue of blockade of cell proliferation, WNT-TCF pathway responses, and tumor growth by dominant-active TCF IC_50_ curves of BrdU incorporation for Ivermectin (top row) or Selamectin (bottom row) with the human colon cancer cell line DLD1 and primary Ls174T colon cancer cells. In each graph, there is the superimposition of the response of control (empty vector) and TCF^VP16^ expressing cells to different drug concentrations. Blue arrows highlight the shift in IC_50_ values. Note the complete rescue at low micromolar concentrations for Ivermectin and nanomolar concentrations for Selamectin. Error bars = s.e.m. See Supplementary Fig S6 for results with CC36.Heat maps of the expression of WNT-TCF targets shown as ratios of the values of drug-treated TCF^VP16^ cells over those of drug-treated empty vector cells, for both DLD1 and Ls174T cells, after housekeeping gene normalization. Red: enhancement at or above 1.5-fold. Dark blue: repression at or below 0.6.Western blots showing the repression of CYCLIN D1 protein levels by treatments with Ivermectin (upper panels) or Selamectin (lower panels), plus their rescue by TCF^VP16^ expression. The levels of the housekeeping gene GAPDH are shown as controls.*In vivo* rescue of the inhibitory effects of Ivermectin on DLD1 xenograft growth by enhanced TCF function through expression of dominant-active TCF^VP16^. The arrow indicates the beginning of treatment. Data information: Error bars = s.e.m.*n* = number of tumors per condition.*P*-values are from*t*-tests.

To further investigate the specificity of Ivermectin and Selamectin for the WNT-TCF pathway, we attempted to rescue their effects in colon cancer cells by directly enhancing TCF function via TCF^VP16^, a fusion of the TCF DNA-binding domain plus the strong VP16 viral transcriptional transactivator (Kim*et al*, 2000) that acts in a WNT- and β-CATENIN-independent manner (Supplementary Fig S6). TCF^VP16^-expressing cells were insensitive to low concentrations of Ivermectin or Selamectin*in vitro* in BrdU incorporation assays, with full rescue observed at 0.5–1 μM for Ivermectin and at 0.05–0.1 μM for Selamectin as compared with controls in DLD1, Ls174T, and primary TNM3 CC36 cells (8) (Fig5A, Supplementary Fig S6). At higher concentrations, these drugs likely engage additional mechanisms (see Discussion).

TCF^VP16^ expression led to strongly boosted positive WNT-TCF target gene levels in colon cancer cells, shown as ratios of those in Ivermectin-treated TCF^VP16^ cells over those in Ivermectin-treated control cells (Fig5B). Low concentrations of Ivermectin (1 μM) or Selamectin (0.1 μM) also repressed the normal levels of CYCLIN D1 protein by 90 and 50%, respectively, detected in DMSO-treated controls, and this repression was fully reversed by expression of TCF^VP16^ (Fig5C).

Importantly, sustained expression of TCF^VP16^ through integrated lentivectors in DLD1 cells rescued the growth blockade of pre-established xenografts by Ivermectin (Fig5D).

### Ivermectin and Selamectin decrease the levels of C-terminal phosphoforms of β-CATENIN and the levels of the direct TCF target CYCLIN D1

**Figure 6:**
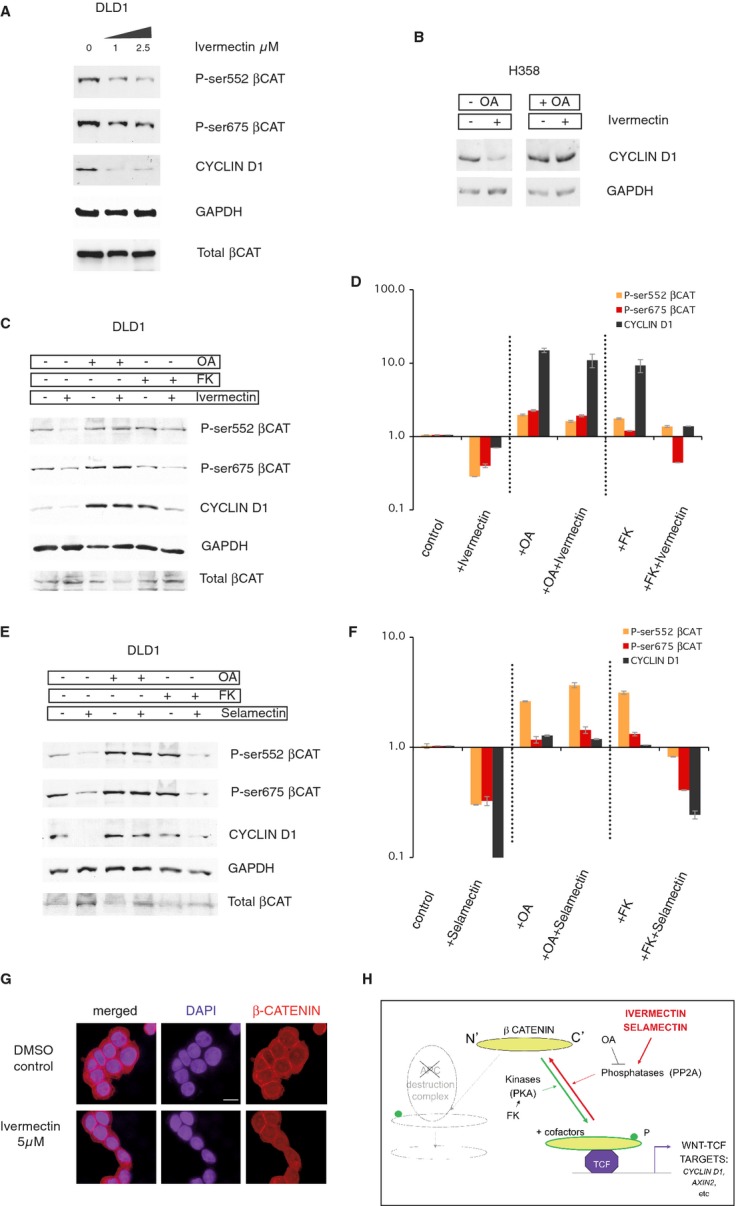
Repression of C-terminal phosphoforms of β-CATENIN and of CYCLIN D1 by Ivermectin and Selamectin and their rescue by phophatase inhibition A Western blot of DLD1 human colon cancer cell extracts showing the concentration-dependent loss of C-terminal phosphoforms of β-CATENIN (P-Ser552 and P-Ser675), and of the levels of the TCF target CYCLIN D1, by Ivermectin at 1 and 2.5 μM. GAPDH and total β-CATENIN levels are shown as controls. B The repressive effect of Ivermectin (5 μM) on CYCLIN D1 levels, used here as a signature for final TCF output, is also observed in lung cancer H358 cells, and this is rescued by okadaic acid (15 nM) treatment. C–F Western blots (C, E) and their quantification (D, F) showing the rescue of the inhibitory effect of Ivermectin (5 μM) (C, D) and Selamectin (0.5 μM) (E, F) by OA (15 nM) but not by FK (10 μM) treatment. GAPDH levels are shown as loading controls in all panels. All treatments were for 12 h to highlight early responses. G Subcellular localization of total β-CATENIN protein in control CC14 cells (top) and in those treated with Ivermectin (5 μM) for 6 h. The panels show confocal microscopy images for β-CATENIN (right), DAPI highlighting the nuclei (center) and merged images (left). Scale bar = 15 μm for all panels in (G). H Diagram of the transition of C-terminally unphosphorylated β-CATENIN to its phosphorylated version, which with TCF and co-factors activate the transcription of target genes including notably that of CYCLIN D1. Phosphorylation is promoted by protein kinases such as Protein Kinase A (PKA), which is activated by Forskolin (FK), and inhibited by phosphatases such as PP2A that is inhibited by okadaic acid (OA). The action of Ivermectin and Selamectin is thus suggested to require active phosphatases. Data information: Error bars = s.e.m.

Since Ivermectin repressed TCF reporter activity driven by APC-insensitive N'Δβ-CATENIN, we sought to test whether this drug could affect downstream activation of β-CATENIN/TCF, focusing on C-terminal phosphorylation of β-CATENIN itself, which is required for full TCF complex transcriptional activity (Hino*et al*, 2005; Fang*et al*, 2007; Zhao*et al*, 2010). Treatment of DLD1 and Ls174T colon cancer cells*in vitro* with Ivermectin led to a dose-dependent inhibition of the levels of two C-terminal phosphoforms of β-CATENIN: Phospho-Ser552 and Phospho-Ser675 and to the repression of CYCLIN D1 (Fig6A, Supplementary Fig S7). GAPDH and total β-CATENIN levels were unchanged (Fig6A, Supplementary Fig S7).

### The inhibitory activity of Ivermectin and Selamectin on CYCLIN D1 levels requires the function of serine protein phosphatases

Decreased levels of β**-**CATENIN phosphoforms could, in principle, result from the inhibition of kinases that phosphorylate C-terminal sites or from the superactivation of phosphatases that dephosphorylate these sites. Therefore, to test for an effect of Ivermectin in promoting the loss of C-terminal β-CATENIN phosphorylation, we first assayed for a possible rescue by okadaic acid (OA), which blocks the activity of the serine protein phosphatases PP2A and PP1. As expected, treatment with OA led to elevated levels of β-CATENIN C-terminal phosphoforms and of the TCF target CYCLIN D1 (Supplementary Fig S8). However, Ivermectin was ineffective in reducing OA-enhanced expression (Fig6C and D, Supplementary Fig S8) as in controls (Fig6A,C and D). Similar results for CYCLIN D1 were also obtained in lung cancer H358 cells (Fig6B). These results suggest that Ivermectin requires active PP2A/PP1 to exert its repressive effect on the WNT-TCF pathway.

To complement these findings, we sought to superactivate the WNT-TCF pathway by enhancing kinase activity through the activation of endogenous Protein Kinase A, which phosphorylates β-CATENIN at C-terminal sites (Hino*et al*, 2005), with forskolin (FK). Treatment with FK was unable to rescue the inhibitory effect of Ivermectin on the levels of C-terminal phosphoforms of β-CATENIN and of CYCLIN D1 (Fig6C and D), indicating that, unlike hyperphosphorylation by phosphatase inhibition, hyperphosphorylation via enhanced kinase activation is subject to repression by Ivermectin.

Finally, these effects were fully reproduced by Selamectin, where OA but not FK, rescued its effects (Fig6E and F), suggesting a similar mode of action for these two macrocyclic lactones on WNT-TCF signaling (Fig6H).

### Localization of β-CATENIN after Ivermectin treatment

As a further test for the action of Ivermectin on β-CATENIN, we mapped its subcellular distribution by immunocytochemistry. Analyses of multiple samples showed similar distributions in control (DMSO) and Ivermectin (5 μM)-treated CC14 cells 6 h after treatment (Fig6G). In both cases membrane, cytoplasmic and nuclear signals were detected.

## Discussion

Here we report that Ivermectin (Campbell*et al*, 1983), an off-patent drug approved for human use, and related macrocyclic lactones, have WNT-TCF pathway response blocking and anti-cancer activities. Whereas the exact molecular target for Ivermectin and Selamectin that affects WNT-TCF responses remains to be identified, the present findings show that these drugs block WNT-TCF pathway responses, likely acting at the level of β**-**CATENIN/TCF function, affecting β**-**CATENIN phosphorylation status.

The similar anti-proliferative activities of Abamectin, Doramectin, and Moxidectin with those of Ivermectin and Selamectin suggest that macrocyclic lactones of the Avermectin and Milbemycin families share common properties and structural features (e.g., Awasthi*et al*, 2012) that may be the basis of the anti-WNT-TCF activities of Ivermectin and Selamectin. Moreover, the potent WNT-TCF response inhibitory activity of Selamectin strongly argues that the activity of macrocyclic lactone preparations made through fermentation, such as Ivermectin, is not due to secondary or residual components since Selamectin is a semi-synthetic single compound drug. The finding that Selamectin is tenfold more potent begs its clinical testing but also the further exploration of the macrocyclic lactone chemical space.

Ivermectin has a well-known anti-parasitic activity mediated via the deregulation of chloride channels, leading to paralysis and death (Hibbs & Gouaux, 2011; Lynagh & Lynch, 2012). The same mode of action has been suggested to underlie the toxicity of Ivermectin for liquid tumor cells and the potentiation or sensitization effect of Avermectin B1 on classical chemotherapeutics (Drinyaev*et al*, 2004; Sharmeen*et al*, 2010). In contrast, the specificity of the blockade of WNT-TCF responses we document, at low micromolar doses for Ivermectin and low nanomolar doses for Selamectin, indicate that the blockade of WNT-TCF responses and chloride channel deregulation are distinct modes of action. In support of this, WNT-TCF response blocking activity is detected at up to tenfold lower concentrations than those reported for chloride ion deregulation (this work; Drinyaev*et al*, 2004). The finding that Moxidectin is more potent than Ivermectin in controlling intestinal nematodes (Fatima*et al*, 2007; Cringoli*et al*, 2009) but similarly or less active on human cancer cells (this work), further supports different modes of action of macrocyclic lactones on cancer cells vs. parasites. The specificity and selectivity of Ivermectin and Selamectin we describe are also inconsistent with ubiquitous effects of these macrocyclic lactones through alterations of anion-selective Cys-loop channels (Hibbs & Gouaux, 2011; Lynagh & Lynch, 2012) or Farnesyl X receptors (Jin*et al*, 2013). The idea that Ivermectin can target different molecules is further supported by its inhibition of flaviviral helicase activity present only during viral replication in infected cells (Mastrangelo*et al*, 2012;). What is key then is to find a dose and a context where the use of Ivermectin has beneficial effects in patients, paralleling our results with xenografts in mice.

Cell toxicity appears at doses greater (> 10 μM for 12 h or longer or > 5 μM for 48 h or longer for Ivermectin) than those required to block TCF responses and induce apoptosis. General toxicity related to chloride channel deregulation has been suggested to underlie the high micromolar toxicity of Ivermectin for liquid tumor cells*in vitro* (Drinyaev*et al*, 2004; Sharmeen*et al*, 2010) and might complicate the treatment of WNT-TCF-dependent brain diseases since Ivermectin can affect glutamate-gated and other Cys-loop ion chloride channels (Kokoz*et al*, 1999; Hibbs & Gouaux, 2011; Lynagh & Lynch, 2012). Ligand-gated chlorine channels are found in the mammalian central nervous system and are normally protected from systemic Ivermectin treatments by the blood–brain barrier (Schinkel*et al*, 1994), but this is often broken in brain tumors.

Our data point to a repression of WNT-β**-**CATENIN/TCF transcriptional responses by Ivermectin, Selamectin and related macrocylic lactones. This conclusion is based on (i) The ability of Avermectin B1 to inhibit the activation of WNT-TCF reporter activity by N-terminal mutant (APC-insensitive) β**-**CATENIN as detected in our screen; (ii) The ability of Avermectin B1, Ivermectin, Doramectin, Moxidectin and Selamectin to parallel the modulation of WNT-TCF targets by dnTCF; (iii) The finding that the specific WNT-TCF response blockade by low doses of Ivermectin and Selamectin is reversed by constitutively active TCF; (iv) The repression of key C-terminal phospho-isoforms of β**-**CATENIN resulting in the repression of the TCF target and positive cell cycle regulator CYCLIN D1 by Ivermectin and Selamectin; (v) The specific inhibition of*in-vivo*-TCF-dependent, but not*in-vivo*-TCF-independent cancer cells by Ivermectin in xenografts.

Analyses of phospho-isoforms of β**-**CATENIN after treatment with Ivermectin or Selamectin under PP2A/PP1 protein phosphatase-blocked conditions suggest that these drugs may act by enhancing, directly or indirectly, phosphatase activity involved in dephosphorylating P-Ser552/P-Ser675. This effect can help explain the phenotype of Ivermectin-treated cells since P-Ser552- and P-Ser675-β-CATENIN show enhanced transcriptional activity in cooperation with TCF factors and are essential for WNT signaling in colon cancer cells (Hino*et al*, 2005; Taurin*et al*, 2006; Fang*et al*, 2007; Zhu*et al*, 2012a). Support for an involvement of PP2A also derives from the finding that its Bα (PR55α) subunit is required to downregulate the levels of P-Ser552 and P-Ser675 C-terminal phosphoforms of β**-**CATENIN in colon cancer cells (Zhang*et al*, 2009). The role of PP2A and its multiple subunits is thus likely to be complex since it can also act positively on WNT signaling with its B56 subunit through the inhibition of N-terminal β**-**CATENIN phosphorylation and thus inhibition of β**-**CATENIN destruction (Seeling*et al*, 1999). Nevertheless, in cancer cells lacking APC destruction complex function (e.g. lacking APC), the overall effect of enhancing PP2A function via Ivermectin treatment is predicted to be pathway silencing (Fig6H).

The elucidation of the effects of Ivermectin discussed above benefited from the fact that most human colon cancer cells tested*in vitro* in monolayers are TCF-dependent (e.g., van de Wetering*et al*, 2002; Varnat*et al*, 2010), thus allowing the*in vitro* screen. Interestingly, the capacity of cancer cells to form 3D spheroids in culture, as well as the growth of these, is also WNT-TCF-dependent (Kanwar*et al*, 2010) and they were also affected by Ivermectin treatment. These results together with the reduction of the expression of the colon cancer stem cell markers*ASCL2* and*LGR5* (e.g., Hirsch*et al*, 2013; Ziskin*et al*, 2013) raise the possibility of an inhibitory effect of Ivermectin, Selamectin and related macrocyclic lactones on TCF-dependent cancer stem cells.

*In vivo*, our previous work has shown that most colon cancer tested in xenografts are TCF-independent. For example, Ls174T and primary CC14 cells are TCF-dependent*in vitro* but become TCF-independent in xenografts*in vivo* (and vice versa) (Varnat*et al*, 2010). In contrast, DLD1 remain TCF-dependent*in vitro* and*in vivo* (Varnat*et al*, 2010). The basis for these changes remains unclear although DNA methylation might be involved (de Sousa*et al*, 2011). Notwithstanding the mechanism, these differences afforded a key test for the specificity of Ivermectin*in vivo*. If Ivermectin is specific, it should only block TCF-dependent tumor growth. Indeed, the sensitivity and insensitivity of DLD1 and CC14 xenografts to Ivermectin treatment, respectively, together with the desensitization to Ivermectin action*in vivo* by constitutively active TCF provide evidence of the specificity of this drug to block an activated WNT-TCF pathway in human cancer.

Ivermectin has a good safety profile since only*in-vivo*-dnTCF-sensitive cancer xenografts are responsive to Ivermectin treatment, and we have not detected side effects in Ivermectin-treated mice at the doses used. Whereas it remains likely that higher doses may begin to show non-specific toxicity, previous work has shown that side effects from systemic treatments with clinically relevant doses in humans are rare (Yang, 2012), that birth defects were not observed after exposure of pregnant mothers (Pacqué*et al*, 1990) and that this drug does not cross the blood–brain barrier (Kokoz*et al*, 1999). Similarly, only dogs with mutant*ABCB1* (MDR1) alleles leading to a broken blood–brain barrier show Ivermectin neurotoxicity (Mealey*et al*, 2001; Orzechowski*et al*, 2012).

Oral Ivermectin is already used by millions of people to combat multiple parasite infections, notably through the Mectizan donation program against river blindness (Thylefors, 2008). Given our present data, this drug could therefore be additionally used as a WNT-TCF blocker against different diseases, including multiple WNT-TCF-dependent human cancer types. In this case, this will likely involve a combinatorial approach with standard chemotherapeutics as debulking agents. Indications may include treatment for incurable β-CATENIN/TCF-dependent advanced and metastatic human tumors of the lung, colon, endometrium, and other organs. Ivermectin, Selamectin, or related macrocyclic lactones could also serve as topical agents for WNT-TCF-dependent skin lesions and tumors such as basal cell carcinomas. Moreover, they might also be useful as routine prophylactic agents, for instance against nascent TCF-dependent intestinal tumors in patients with familial polyposis and against nascent sporadic colon tumors in the general aging population. Formulations of Ivermectin and other hydrophobic macrocyclic lactones with agents that enhance tissue penetration may improve their efficacy.

## Materials and Methods

### Cell culture

Human colon cancer CC14 and CC36 primary cells and Ls174T and HT29 cell lines were cultured as previously described (Varnat*et al*, 2009, 2010). Colon cancer DLD1 and lung non-small cell bronchioalveolar carcinoma H358 cells were cultured as attached 2D layers in DMEM-F12 or RPMI 1640 with 10% FBS. 293T cells were cultured in DMEM with 10% FBS. For drug treatments, cells were plated in 2% FBS and treated the next day for 12–48 h. GBM primary cells were cultured as described in Zbinden*et al* (2010). U251 glioma and SKMel2 melanoma cell lines were cultured as described by ATCC.

### Primary screening

A luciferase reporter assay was performed in 293T cells to identify compounds able to decrease TCF-driven gene expression using a TCF-binding site fused to a firefly luciferase reporter construct. Activation was driven by transfected N'Δβ-CATENIN acting on endogenous TCF factors. Internal controls derived from Renilla luciferase activity resulting from a HSV-TK expression plasmid co-transfected in all cases. Transfection of 293T cells was preformed with calcium phosphate and plated at a density of 5,000/well into 96-well plates. Each transfection combined a constitutively active β-CATENIN plasmid as effector, a TCF-binding site firefly luciferase plasmid as reporter, and a HSV-TK Renilla luciferase plasmid as control. Cells were harvested 12 h after compounds treatment. Secondary reporter assays were repeated at least three independent times.

### Drugs

Abamectin (31732, Fluka), Ivermectin (Fagron Iberica, and Sigma #I8898), Doramectin (33993, Fluka), Selamectin (32476, Sigma), Stromectol™ (Merck) and Moxidectin (33746, Sigma) were diluted in 100% DMSO or ethanol and used at 0.01–10 μM for*in vitro* assays. Okadaic acid (OA, Enzo Life Science) was used at 1–15 nM.

### BrdU incorporation

Colon primary cell culture and cell lines were plated in medium containing 2% FBS and treated with different drug concentrations for 48 h. After a 15′ pulse with BrdU (10 mg/ml), cells were fixed in 4% PFA, incubated in 2N HCl for 15′, neutralized with 0.1 M Boric acid for 10′, blocked in PBS-10% HINGS for 10′, and labeled with anti-BrdU antibodies (1:5,000, University of Iowa Hybridoma Bank). Signal was revealed with a rhodamine-coupled anti-mouse secondary antibody (1:500, Invitrogen Molecular Probes) and counterstained with DAPI (Sigma). At least 10 fields were quantified for each condition.

### Apoptosis assay

Colon cancer cells were plated at 30% confluency and treated with different drug concentrations for 48 h. After treatment, cells were fixed in 4% PFA for 2 min and blocked in 10% HINGS for 1 h. Apoptosis was evaluated by immunofluorescence using a rabbit anti-cleaved Caspase3 antibody (1:200, overnight at 4°C; Cell Signaling) and Cy3-labeled secondary antibody (1:500, 1 h at RT; Jackson ImmunoResearch). Cells were counterstained with DAPI (1:10,000, Sigma). At least 10 fields were quantified per plate for each condition.

### Quantitative reverse transcription PCR

RNA extraction, reverse transcription and qPCR were performed as previously described (Varnat*et al*, 2009).

qPCR primer sequences were as follows (5′->3′):

**Table:** 

LGR5-F	GGAGCATTCACTGGCCTTTA
>LGR5-R	CTGGACGGGGATTTCTGTTA
P21-F	GACTCTCAGGGTCGAAAACG
P21-R	AAGATGTAGAGCGGGCCTTT
AXIN2-F	CTCCTTATCGTGTGGGCAGT
AXIN2-R	CCAACTCCAGCTTCAGCTTT
EFNB1-F	AAGAACCTGGAGCCCGTAT
EFNB1-R	GGGGTCGAGAACTGTGCTAC
EPHB2-F	ATGCGGAAGAGGTGGATGTA
EPHB2-R	CCTTGAAAGTCCCAGATGGA
EPHB3-F	GATTGGCTGAAGTACCAGACC
EPHB3-R	CAGAAACTGCAGACTCCTGG
OLFM4-F	GGACTTCGAGCTGATCAAGG
OLFM4-R	CGACAGGGGTGTTTTGATCT
SOX4-F	AAACCAACAATGCCGAGAAC
SOX4-R	GTTCATGGGTCGCTTGATGT
ASCL2-F	GCGTTCCGCCTACTCGT
ASCL2-R	GGCTTCCGGGGCTGAG
TBP-F	TGCACAGGAGCCAAGAGTGAA
TBP-R	CACATCACAGCTCCCCACCA
HMBS-F	AAGTGCGAGCCAAGGACCAG
HMBS-R	TTACGAGCAGTGATGCCTACCAAC
cMYC-F	TGGTCTTCCCCTACCCTCT
cMYC-R	GATCCAGACTCTGACCTTTT

### *In vitro* clonogenic assays and CD133 sorting

Adherent colon cancer cells were treated with Ivermectin or Selamectin at different concentrations as described, washed, lifted, and plated at 1 cell/well in 96-well non-adherent plates in colon spheroid medium (DMEM-F12 supplemented with B27 and 10 ng/ml EGF) without drugs and grown for 14 days. Spheres were then counted visually using an inverted Zeiss optical microscope. For secondary clonogenic assays, primary spheres were dissociated and replated at 1 cell/well in 96-well plates and cultured as described above. Each experiment was repeated at least thrice. Alternatively, untreated cells were plated and treated in 96-well plates with drugs and subsequently scored for spheroid formation. CD133 MACS (Miltenyi Biotech) was performed as described previously (Varnat*et al*, 2009, 2010) using either limited Trypsin or StemProAccutase (Gibco LifeTech) for cell dissociation.

### Western blots

Cells were plated in media containing 2% FBS and treated with Ivermectin or Selamectin at different concentrations for 12 h. Protein lysates were in RIPA buffer. 10–20 μg of protein were loaded onto 12% polyacrylamide gels and the proteins transferred onto nitrocellulose membranes (Hybond), which were probed with primary and secondary antibodies as noted. Signal was revealed using the ECL system (GE Healthcare) and film. ImageJ was used for quantification. Membranes were sequentially reprobed after washing in TBS-1% Tween-20 and controlling for loss of signal. The following primary antibodies were used: anti-GAPDH (1:6,000 for 1 h at RT, 2118 Cell Signaling), anti-cyclinD1 (1:100 for 1 h at RT, Ab-3 Oncogene research), anti-Pser552 β-CATENIN (1:1,000 for 1 h at RT, 9566 Cell Signaling), anti-Pser675 β-CATENIN (1:1,000 for 1 h at RT, 4176 Cell Signaling), and anti-total β-CATENIN (1:1,000 for 1 h at RT, 8480 Cell Signaling). As secondary antibodies, HRP-coupled anti-rabbit or anti-mouse (1:6,000, Promega) were incubated for 1 h at RT.

### Mouse xenografts

5 × 10^5^ control or lentivector-infected cancer cells were injected subcutaneously into the flanks of 6- to 8-week-old female NMRI Nude mice. After tumor establishment, mice were treated with cyclodextrin carrier alone or Ivermectin (or Stromectol™) conjugated with cyclodextrin (45%) via daily intraperitoneal injections at 10 mg/kg. Tumor volumes were measured every 2–3 days.

### Sequencing*APC* and*β-CATENIN* commonly mutated regions

One-hundred nanograms of genomic DNA from primary colon cancer cells (CC14 and CC36) or cell lines (DLD1 and Ls174T) was used for PCR using Phusion HF DNA Polymerase (BioLabs). Specific primers were designed to amplify commonly mutated regions in*APC* and*CTNNB1* (*β-CATENIN*) genes. The Mutation Cluster Region (MCR) of*APC* was amplified with two pairs of primers: (1) MCR1FWD 5′-GATACTCCAATATGTTTTTC-3′ and MCR1REV 5′-GGAAGATCACTGGGGCTTAT-3′, (2) MCR2FWD 5′-GTGAACCATGCAGTGGAATG-3′ and MCR2REV 5′-TCTGAATCATCTAATAGGTC-3′. The primers for*CTNNB1* were as follows:*CTNNB1*FWD 5′-CAATGGGTCATATCACAGATTCTT-3′ and*CTNNB1*REV 5′-TCTCTTTTCTTCACCACAACATTT-3′.

**The paper explained**

**Problem**

The WNT-TCF intercellular signaling pathway is constitutively activated in a large number of diseases, including cancers of the intestine, skin, lung, endometrium, and brain. Finding small molecules that would act as pathway blockers has attracted much therapeutic interest but so far there are no available WNT-TCF blockers in the clinics, partly due to the time and cost of developing new drugs.

**Results**

Here, we have performed a repositioning small molecule screen using a collection of clinically approved drugs. We describe the identification of Ivermectin and related macrocyclic lactones, including the single-component semi-synthetic drug Selamectin, as WNT-TCF blockers in human cancer cells*in vitro* and*in vivo*. Selamectin is 10 times more potent than Ivermectin, acting in the nanomolar range. Low doses of these drugs mimic the effect of dominant-negative TCF and their actions are rescued by activated TCF (TCF^VP16^). Ivermectin shows exquisite selectivity*in vivo*, repressing TCF-sensitive human cancer xenograft growth with no discernable side effects at the doses used. WNT-TCF blocking activity is detected at doses below those reported for chloride channel deregulation and general cell toxicity, suggesting a therapeutic window to target hyperactivated WNT-TCF-dependent diseases.

**Impact**

Given that Ivermectin is an approved drug widely used to combat multiple parasitic infections, our data suggest its additional use as a therapeutic agent against WNT-TCF-dependent diseases, notably including several cancer types, and the further investigation of the macrocyclic lactone chemical space.

## β-CATENIN immunohistochemistry

Colon cancer cells were plated at 40% confluency and treated with 5 μM Ivermectin or 2% DMSO for 6 h. After treatment, cells were fixed in fresh 4% PFA for 2 min. Antigen retrieval was conducted by boiling for 7 min in 10 mM citrate buffer (10 mM citric acid, 0.05% Tween-20, pH 6.0). Cells were then incubated at RT for 30 min, washed with PBS and permeabilized in PBS with 1% Tween-20 for 15 min at RT. Anti-β-CATENIN antibodies (1:400, Cell Signaling) were applied overnight at 4°C after blocking with 3% BSA in PBS. Signal was revealed with a rhodamine-coupled anti-rabbit secondary antibody (1:1,000) and counterstained with DAPI (1:10,000). Samples were imaged with Zeiss LSM 510META confocal microscope. Five independent slides were analyzed per each condition.
