# Supplementary material for: The river blindness drug Ivermectin and related macrocyclic lactones inhibit WNT-TCF pathway responses in human cancer
Source: EMBO Mol Med. 2014 Aug 20;6(10):1263–78. doi: 10.15252/emmm.201404084 (PMC4287931; doi:10.15252/emmm.201404084)
Supplement: Supplementary file 1 [file emmm0006-1263-sd1.pdf]

Supporting Information online

THE RIVER BLINDNESS DRUG IVERMECTIN AND RELATED MACROCYCLIC LACTONES INHIBIT WNT-TCF PATHWAY RESPONSES IN HUMAN CANCER

Alice Melotti•, Christophe Mas•, Monika Kuciak•, Aiala Lorente-Trigos, Isabel Borges and Ariel Ruiz i Altaba*

University of Geneva, Dept. of Genetic Medicine and Development, 8242 CMU, 1 rue Michel Servet, CH-1211 Geneva, Switzerland

•equal first authorship

*To whom correspondence should be addressed: +41-22-379-5646

Ariel.RuizAltaba@unige.ch

Supporting Figures

Figure S1. Luciferase assays of the top 9 hits.

Representative Luciferase reporter assays in 293T cells performed in triplicates with the top 9 antagonist candidates, confirming the results of the screen. Histogram shows averages with error bars representing s.e.m.

Figure S2. Gene expression and anti-proliferative effects of top hit 4B5 in various colon cancer cells.

A) Histogram of percentage changes of experimental gene expression over DMSO control for the genes shown after normalization by housekeeping gene levels in Ls174T and primary human colon cancer CC14 cells with 4B5 at 10μM 12h post-treatment. Actual values are indicated above the columns. All changes are significative over controls (T-test $p<0.05$).

B) Histogram of the quantification of the effects of different concentrations of compound 4B5 on BrdU incorporation in the human colon cancer cell line Ls174T and the primary human colon cancer CC14 and metastatic mCC11 cells. Values are percentage incorporation equating the levels of control DMSO-treated cells to 100%. Asterisks denote significative changes over control (T-test $p<0.05$)

Error bars = s.e.m.

Figure S3. Structures and activities of selected macrocyclic lactones.

A,D) Name, composition and chemical structure of macrocyclic lactones. Note mixtures are denoted by the varying R group. Major changes in structures are highlighted with red circles.

B,C,E) Tables of percentage BrdU incorporation shown as averages of triplicates normalized over DMSO controls (equated to 100%) over a range of concentrations. Calculated IC50's with PRISM are given in the rightmost columns. Stromectol^{TM} is a commercial name of oral Ivermectin used . Results are shown for multiple human cancer types (B,C) and multiple human colon cancer cells (E).

Figure S4. Ivermectin and Selamectin inhibit colon cancer stem cell self-renewal during clonogenic assays.

Histogram of the percentage of spheroid formation in each condition as indicated. Treatment with DMSO (as control) or with Ivermectin at 1μM or 5μM started at the time of clonal plating for spheroid formation.

Error bars = s.e.m. Probability (p) values are derived from 2-tailed T-tests. No p values are possible for comparison with zero values at 5μM.

Figure S5. Inhibitory effects of Stromectol™ and Ivermectin on DLD1 and H358 xenografts, respectively.

A) Graph of tumor growth in control or Stromectol-treated mice bearing DLD1 xenografts. See Figure 3A for comparison with Ivermectin. In all cases treatment began after tumor establishment. Stromectol™ pills form the local pharmacy were grounded and the powder dissolved in DMSO, then mixed with cyclodextrin as with Ivermectin, aiming at a similar dose (10mg/kg/daily) as given with Ivermectin conjugated with cyclodextrin. We note that the exact amount of active ingredient is likely to vary.

B) Inhibitory effects of Ivermectin on the growth of human metastatic bronchialveolar carcinoma H358 xenografts.

C) Left panel) Heat map of gene expression shown as ratios determined by rt-qPCR of experimental over controls after normalization with housekeeping genes, revealing the repression of WNT-TCF targets by Ivermectin in H358 cells. Right panel) Western blots showing the repression of CYCLIN D1 protein levels by Ivermectin treatment in H358 cells. Control GAPDH levels are shown for each condition.

Error bars = s.e.m. Probability (p) values are derived from 2-tailed T-tests.

Figure S6. Diagram of direct activation of TCF by the fusion protein TCF^{VP16} and IC50 curves for treated Ls174T cells.

A) Diagram of the suggested action of macrocyclic lactones on β-CATENIN-TCF-driven transcription of targets and its rescue by the function of a TCF-VP16 transactivating domain fusion that acts as a dominant-active TCF independent of β-CATENIN.

B) IC50 curves of BrdU incorporation for Ivermectin (left) or Selamectin (right) treatments with primary human colon cancer CC36 cells. In each graph there is the superimposition of the response of control (empty vector) and TCF^{VP16} expressing cells to different drug concentrations. Blue arrows show the shift in IC5O values. Error bars = s.e.m. See Figure 5A.

Figure S7. Repression of C-terminal phosphoforms of β-CATENIN and CYCLIN D1 by Ivermectin in Ls174T cells.

Western blots of Ls174T human colon cancer cell extracts showing the concentration-dependent loss of C-terminal phosphoforms of β-CATENIN (P-ser552 and P-ser675) and CYCLIN D1 by treatments with Ivermectin. See Figure 6. GAPDH and total β-CATENIN levels are shown as controls.

Figure S8. Okadaic acid effects and rescue of Ivermectin repression of C-terminal phosphoforms of β-CATENIN and CYCLIN D1.

A) Dose-dependent increases of C-terminal phosphoforms of β-CATENIN and of the levels of CYCLIN D1 in DLD1 cells treated with Okadaic acid (OA).

B) Western blots of DLD1 cell extracts showing varying levels of C'-phosphoforms of β-CATENIN and of CYCLIN D1 treated with (+) 15nM Okadaic acid (OA) as compared with DMSO only (-) controls. Treatment with OA, which represses the activity of the protein phosphatases PP2A and PP1, rescues the repression of these proteins by Ivermectin treatment (2.5μM and 5μM, gray triangle). The control and experimental lanes are from the same Western blot (see Source data for FigS8B).

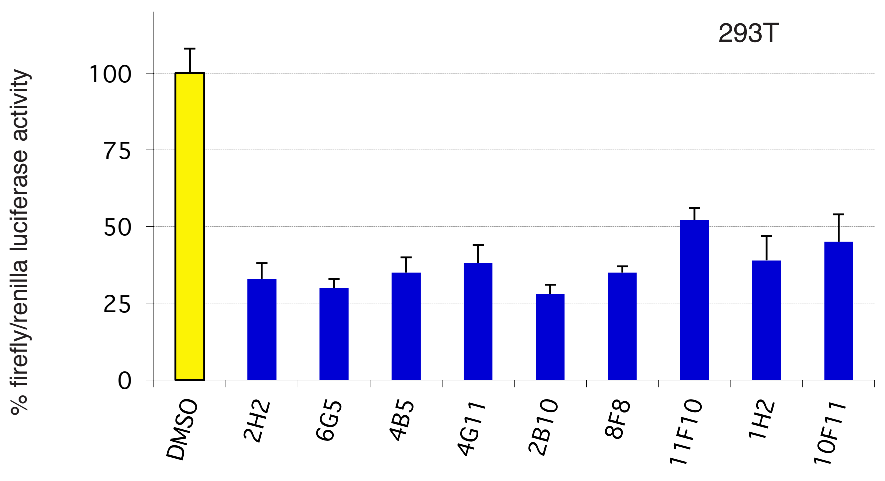
293T
% firefly/renilla luciferase activity
100
75
50
25
0
DMSO
2H2
6G5
4B5
4G11
2B10
8F8
11F10
1H2
10F11

Fig S1

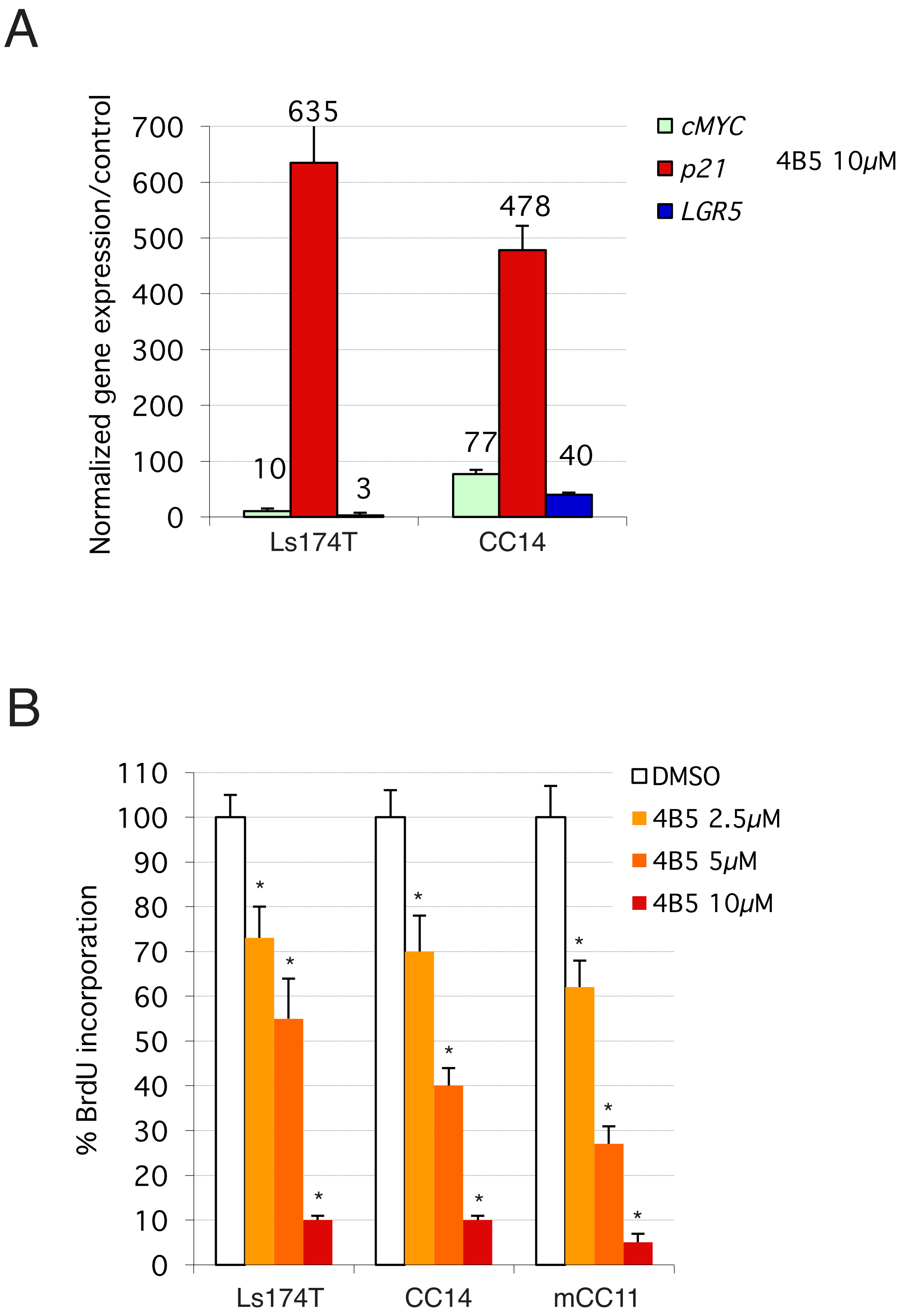
A
Normalized gene expression/control
700
600
500
400
300
200
100
0
635
478
10
3
77
40
Ls174T
CC14
cMYC
p21
LGR5
4B5 10μM
B
% BrdU incorporation
110
100
90
80
70
60
50
40
30
20
10
0
Ls174T
CC14
mCC11
DMSO
4B5 2.5μM
4B5 5μM
4B5 10μM

Fig. S2

A

ABAMECTIN = Avermectin B1 = 4B5

> 80% avermectin B_{1a}

+

< 20% avermectin B_{1b}

IVERMECTIN

> 90% 22,23dihydroavermectin B_{1a}

+

< 10% 22,23dihydroavermectin B_{1b}

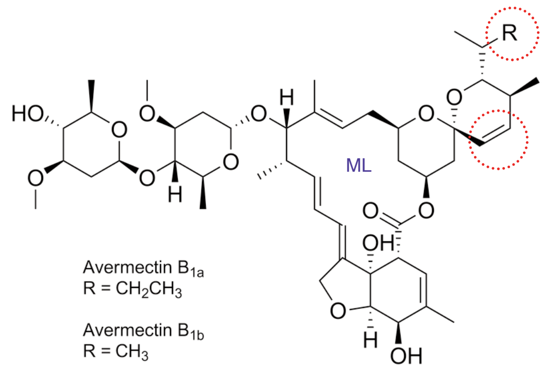

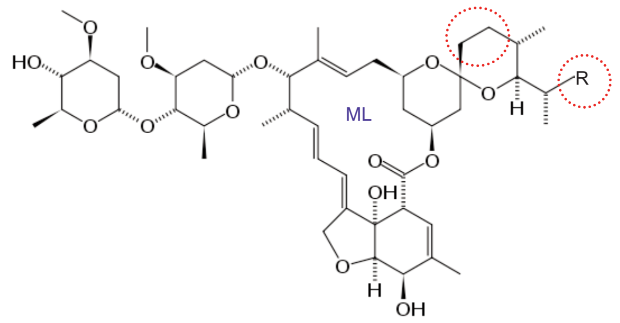

B

% BrdU incorporation/control

		Abamectin (μM) 0.5	1	2.5	5	10	IC50	Ivermectin (μM) 0.5	1	2.5	5	10	IC50
Colon cancer	CC14	70	40	32	0	0	0.8	-	68	57	6	0	2.4
	CC36	75	55	42	10	0	1.4	-	53	14	0	0	1
Glioblastoma	GBM13	100	114	48	0	0	2.4	116	100	16	0	0	1.7

C

% BrdU incorporation/control

		Ivermectin (μM) 1	2.5	5	10	IC50	Stromectol (μM) 1	2.5	5	10	IC50
Colon cancer	DLD1	89	53	15	0	2.6	62	12	7	0	1.2
	CC14	68	57	6	0	2.4	60	35	20	4	1.4
Glioblastoma	U251	96	43	0	0	2.3	39	8	0	0	1.8
	GBM12	95	31	0	0	2.2	108	44	0	0	2
	GBM13	100	16	0	0	1.7	87	19	0	0	2
Melanoma	SKMel2	95	50	0	0	2.5	37	0	0	0	0.8

D

DORAMECTIN

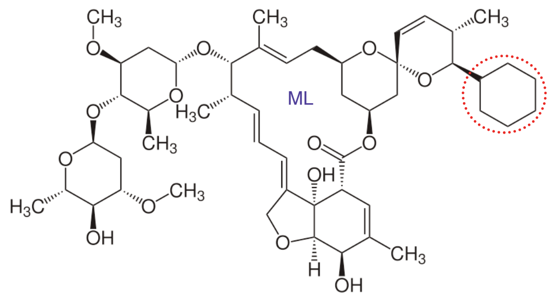

SELAMECTIN

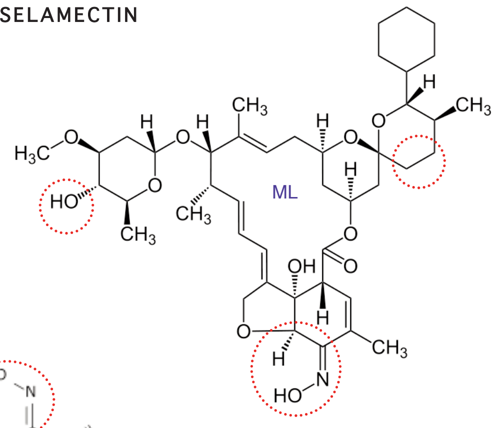

MOXIDECTIN

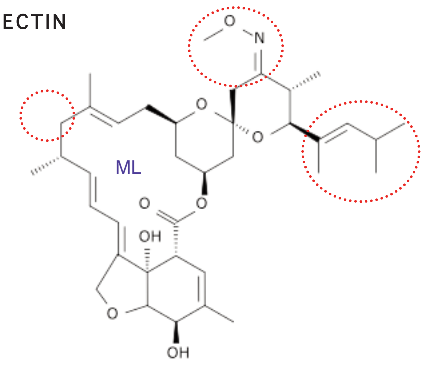

E

% BrdU incorporation/control

	Abamectin (μM) 0.05	0.1	0.5	1	2.5	5	IC50	Ivermectin (μM) 0.05	0.1	0.5	1	2.5	5	IC50
DLD1	117	106	83	93	47	0	2	85	97	86	95	17	0	2.3
LS174T	102	87	79	0	0	0	1	103	101	133	0	0	0	0.8
CC14	90	76	57	41	0	0	1	73	67	34	38	0	0	1

	Doramectin (μM) 0.05	0.1	0.5	1	2.5	5	IC50	Selamectin (μM) 0.01	0.05	0.1	0.5	1	2.5	IC50	Moxidectin (μM) 0.05	0.1	0.5	1	2.5	5	IC50
DLD1	65	92	66	62	56	0	2.8	106	99	79	0	0	0	0.14	96	111	89	61	5	0	1.2
LS174T	88	81	90	0	0	0	0.6	89	64	45	0	0	0	0.08	95	122	112	98	0	0	1.2
CC14	91	87	72	53	0	0	1.1	85	56	56	0	0	0	0.09	94	92	75	79	0	0	1.4

Fig. S3

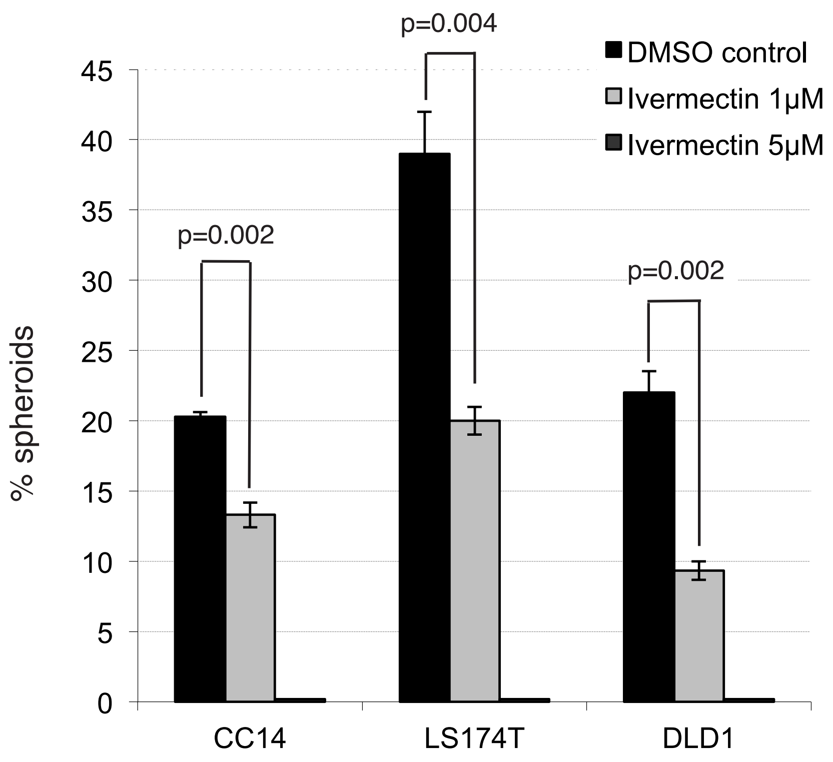
% spheroids
45
40
35
30
25
20
15
10
5
0
CC14
LS174T
DLD1
p=0.002
p=0.004
p=0.002
DMSO control
Ivermectin 1μM
Ivermectin 5μM

Fig. S4

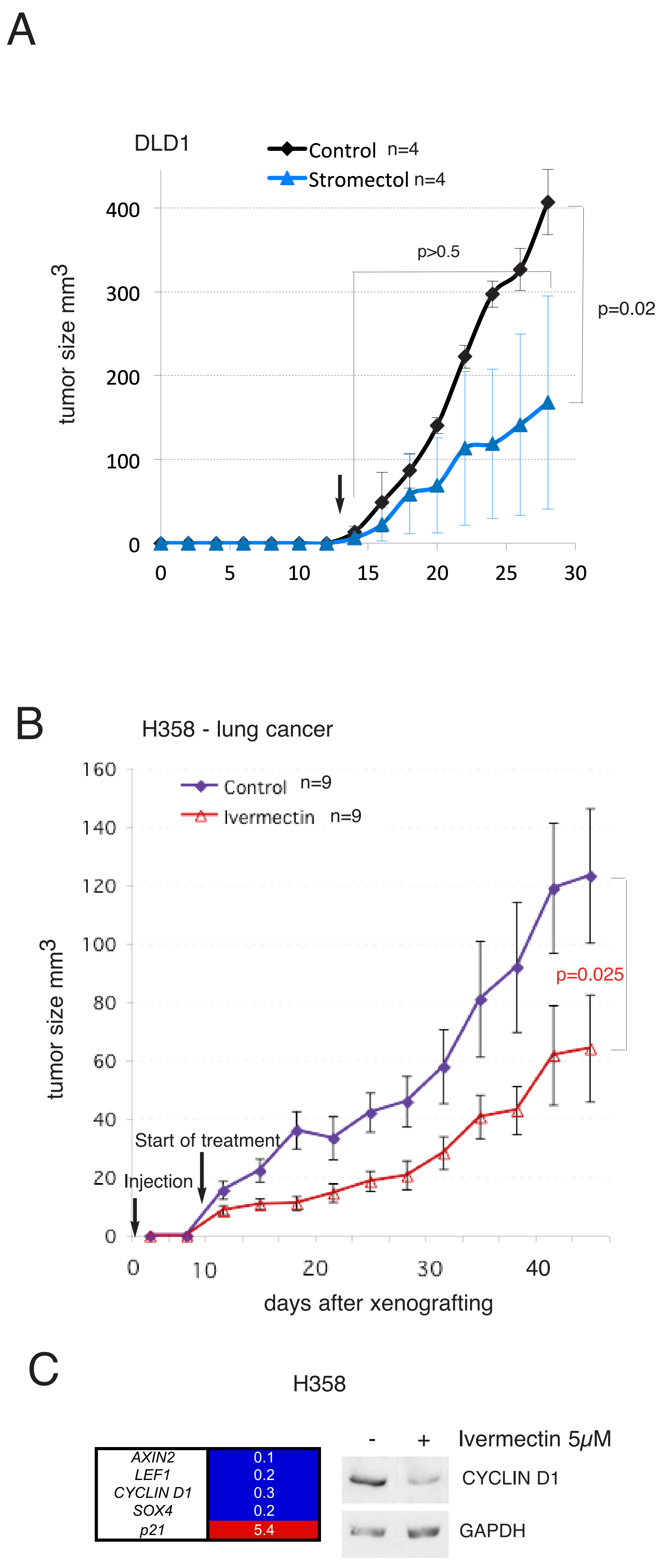
A
DLD1
Control n=4
Stromectol n=4
tumor size mm3
400
300
200
100
0
0
5
10
15
20
25
30
p>0.5
p=0.02
B
H358 - lung cancer
Control n=9
Ivermectin n=9
tumor size mm3
160
140
120
100
80
60
40
20
0
0
10
20
30
40
Start of treatment
Injection
p=0.025
days after xenografting
C
H358
AXIN2 0.1
LEF1 0.2
CYCLIN D1 0.3
SOX4 0.2
p21 5.4
- +
Ivermectin 5μM
CYCLIN D1
GAPDH

Fig. S5

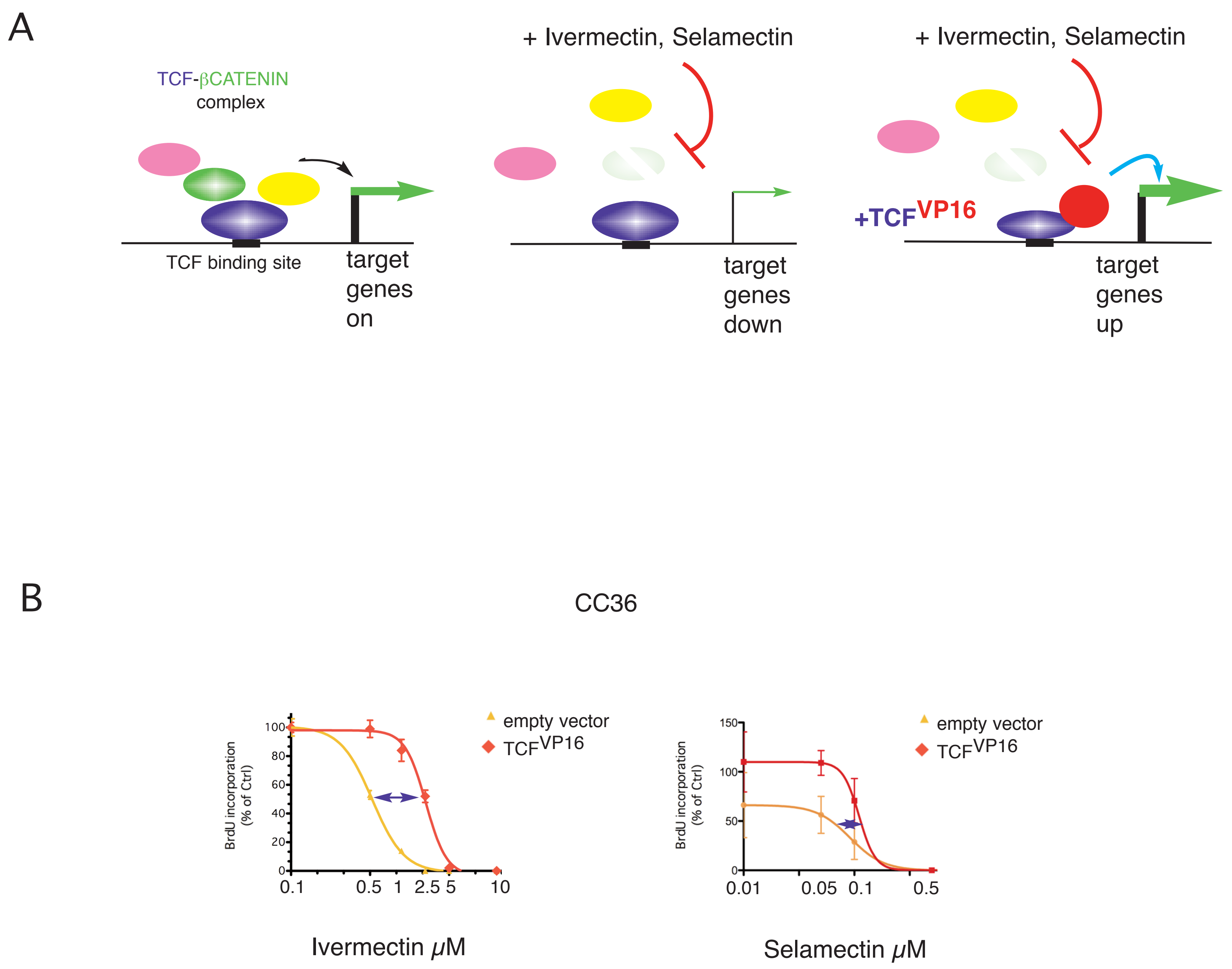
A
TCF-βCATENIN
complex
TCF binding site
target
genes
on
+ Ivermectin, Selamectin
target
genes
down
+ Ivermectin, Selamectin
+TCFVP16
target
genes
up
B
CC36
BrdU incorporation
(% of Ctrl)
empty vector
TCFVP16
0.1
0.5
1
2.5
5
10
Ivermectin μM
0.01
0.05
0.1
0.5
Selamectin μM

Fig. S6

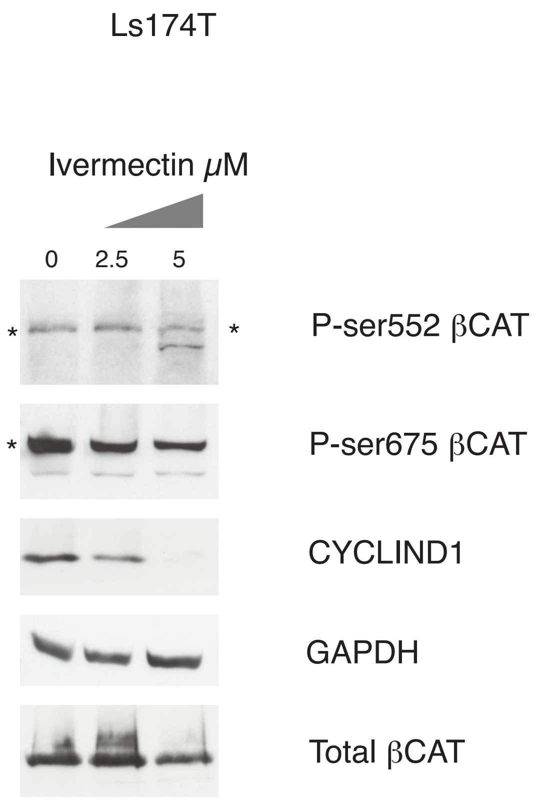
Ls174T
Ivermectin μM
0
2.5
5
*
*
P-ser552 βCAT
*
P-ser675 βCAT
CYCLIND1
GAPDH
Total βCAT

Fig S7

A

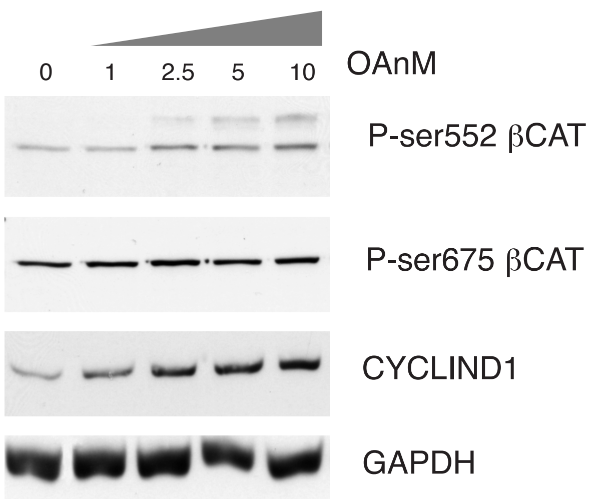

0
1
2.5
5
10
OAnM
P-ser552 βCAT
P-ser675 βCAT
CYCLIND1
GAPDH

B

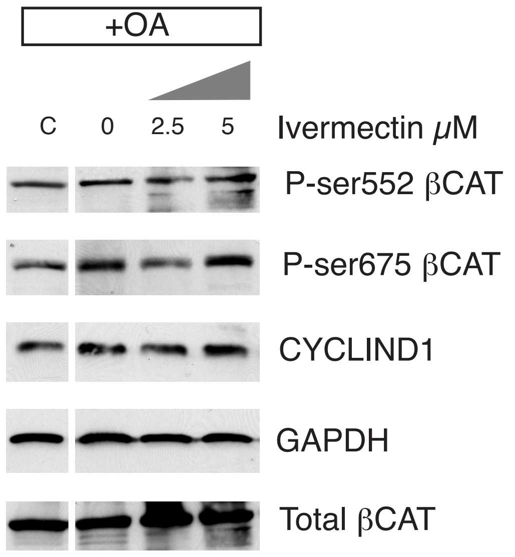

+OA
C
0
2.5
5
Ivermectin μM
P-ser552 βCAT
P-ser675 βCAT
CYCLIND1
GAPDH
Total βCAT

Figure S8
