# Supplementary material for: The river blindness drug Ivermectin and related macrocyclic lactones inhibit WNT-TCF pathway responses in human cancer
Source: EMBO Mol Med. 2014 Aug 20;6(10):1263–78. doi: 10.15252/emmm.201404084 (PMC4287931; doi:10.15252/emmm.201404084)
Supplement: Supplementary file 2 [file emmm0006-1263-sd2.pdf]

EMBO Molecular Medicine

THE RIVER BLINDNESS DRUG IVERMECTIN AND RELATED MACROCYCLIC LACTONES INHIBIT WNT-TCF PATHWAY RESPONSES IN HUMAN CANCER

Alice Melotti, Christophe Mas, Monika Kuciak, Aiala Lorente-Trigos, Isabel Borges and Ariel Ruiz i Altaba

Corresponding author: Ariel Ruiz i Altaba, University of Geneva

Review timeline:

Submission date:	18 March 2014
Editorial Decision:	18 April 2014
Revision received:	26 June 2014
Editorial Decision:	08 July 2014
Revision received:	14 July 2014
Accepted:	15 July 2014

Transaction Report:

(Note: With the exception of the correction of typographical or spelling errors that could be a source of ambiguity, letters and reports are not edited. The original formatting of letters and referee reports may not be reflected in this compilation.)

Editor: Roberto Buccione

1st Editorial Decision 18 April 2014

Thank you for the submission of your manuscript to EMBO Molecular Medicine. We have now heard back from the three Reviewers whom we asked to evaluate your manuscript.

You will see that two Reviewers are more supportive of you work while one is quite negative. All considered, the concerns expressed prevent us from considering publication at this time. I will not dwell into much detail, and just mention a few main points.

Reviewer 1 is fundamentally negative and at multiple levels. S/he clearly does not support publication based on a number of important issues. These include lack of mechanistic insight and details on the impact on cancer stem cells and specific pharmacokinetics and pharmacodynamic studies.

Reviewer 2 is quite positive, but does list a number of technical issues that require your action and consequent clarification.

Reviewer 3 is also positive and suggests a number of improvements that should make your work more compelling. I do note, however, that this Reviewer does suggest further work on the tumour stem cell aspects of the study and suggests that spherogenesis assays would help in defining the inhibition of self-renewal by beta-catenin inhibition. This is part reflects one of the specific issues

mentioned by Reviewer 1.

After extensive discussion and Reviewer cross-commenting, we came to the conclusion that while we agree with Reviewer 1 that a better defined mechanism would make the work more compelling, the significance of the findings appears more important at this stage and potentially very useful to the community. Moreover, the models used appear in line with those in other studies examining colon and lung cancer. In conclusion, provided you deal with comments from reviewers 2 and 3 we would be willing to move forward with your manuscript, with the understanding that the issues must be fully addressed with additional experimentation where appropriate and that acceptance of the manuscript will entail a second round of review. It would be of great interest however, if you could provide more information (beyond speculation) on the general issue of drug penetration vs. impact on tumor initiation vs. maintenance as Reviewer 1 points out.

As you know, EMBO Molecular Medicine has a "scooping protection" policy, whereby similar findings that are published by others during review or revision are not a criterion for rejection. However, I do ask you to get in touch with us after three months if you have not completed your revision, to update us on the status. Please also contact us as soon as possible if similar work is published elsewhere.

I look forward to seeing a revised form of your manuscript as soon as possible.

***** Reviewer's comments *****

Referee #1 (Remarks):

The authors have performed a drug repositioning screen to attempt to identify compounds that might interfere with the WNT-beta catenin pathway. They have identified ivermectin and related compounds as potent inhibitors of signaling downstream of WNT. They have tested with appropriate controls both in vitro and in vivo the activity of the compounds and reasonably conclude that the compounds effects are specific. Unfortunately at this stage the manuscript fails to address a number of essential points and therefore in my opinion is not suitable for publication. The authors might want to consider the following: a. pursue the identification of the molecular target; b. perform detailed pharmacokinetics and pharmacodynamic studies, e.g. Cyclin D1 staining in tissue sections, to also address issues of drug penetration vs. impact on tumor initiation vs. maintenance; c. understand impact on cancer stem cells if any, by performing required customary experiments , e.g. TIC frequency, etc.; d. most importantly, attempt to understand lack of efficacy in vivo, as pursuing these compounds as potential therapeutics needs this question to be solved now rather than later. The manuscript would also benefit from a thorough revision of English style and grammar.

Referee #2 (Remarks):

The work by Melotti, Mas et al. describes a repositioning screening strategy which identifies a class of macrocyclic compounds that can potently inhibit the Wnt signalling pathway. Overall this is an excellent study that has led to the identification of a clinically-experienced, off-patent compound with inhibitory activity. This study will provide a platform for identification of the specific molecular target of Ivermectin and allow further development of more efficacious Wnt signalling pathway inhibitors. I have only minor points to be addressed as follows:
1) Beta-CATENIN is not hyphenated in the intro, but everywhere else. Page 5, pole should read pool (2 instances).

2) Was Ivermectin in the screening library? If so, how did it score in the luciferase reporter assay?

3) Structure-function analyses make use of proliferation assays (BrdU incorporation) and are not complete with respect to specific Wnt pathway activity. Could the authors include luciferase

reporter assay data for Ivermectin and Moxidectin, Bryostatin, Doramecting to allow direct comparison alongside proliferation assays? I realize this is largely covered with target gene analysis however, for direct efficacy comparison between compounds, the relative expression of individual target genes does not appear to be consistent.

4) Are the panels for 3 C, E needed - ie. are there visual differences between the treatments?

5) Ls174T cells harbour the S45 mutation in beta-catenin. Do the primary cells (CC14) which are insensitive to Ivermectin in xenograft assays also harbour mutations in CTNNB1 or are they APC-mutant, similar to the Ivermectin-sensitive DLD1 (and HT-29) cell lines?

6) Is Wnt pathway activity sensitive to Ivermectin with overexpression of the LEF DNA binding domain fused to the C-terminus of beta-CATENIN (such as in [Hao et al (2013) Cell Reports])?

7) The effects of okadaic acid, forskolin and potentially Ivermectin treatment are likely pleitropic and the data implicating the effect of Ivermectin mediated by the action of a phosphatase on C-terminal phosphorylation sites is largely correlative. Moreover, it is not clear from the literature whether the effect of C-terminal phosphorylation affects cellular levels, nuclear localization, the interactions or transcriptional activity of beta-CATENIN. What would be the effect of Ivermectin on expressed versions of beta-CATENIN (or the construct in point 6)) with mutant C-terminal phosphorylation sites (ie. converted to Ala versus Asp)?

Referee #3 (Remarks):

The authors identify and validate commonly used anti-helminthic compounds as novel inhibitors of TCF-beta-catenin activity. The study is highly significant as the Wnt pathway has been implicated in a wide variety of human cancers but few approaches to target this pathway have been developed. Furthermore, many of the compounds are currently under investigation target Wnt signaling at the cell-surface and will likely not be effective in tumor cells that carry mutations or activating events that lie downstream of Fzd receptors. Moreover, the authors' findings that ivermectin and selamectin can inhibit the transcriptional activity of beta-catenin/TCF are highly clinically relevant as these agents are already in clinical use. Overall, the studies are well done and the manuscript is well written.

Although the precise mechanism by which these agents inhibit Wnt signaling is not precisely defined in this manuscript, the authors do a good job demonstrating that the effects of these agents are specific. Furthermore, the main point of the submission, that these common drugs inhibit the Wnt pathway, is significant and well worth reporting.

Minor comments include:

1. The compounds were screened based on the inhibition of proliferation. Given their effects on clonogenic tumor growth in vitro and in vivo, it would be useful to know if either of these compounds also induces apoptosis at the doses that inhibit Wnt target gene expression.

2. For the studies examining clonogenic growth, examination of serial sphere formation would be useful in defining the inhibition of self-renewal by beta-catenin blockade.

3. Similarly, an examination of potential colon cancer CSC marker, such as CD133, may bolster the claims that these cells are undergoing inhibition.

4. In terms of Wnt signaling, was localization of beta-catenin impacted in any way by the compounds?

1st Revision - authors' response 26 June 2014

Please find below a point-per-point reply to the referee's comments. We believe we have replied to all key points and provide additional data to support our findings and claims. As such we feel our paper should now be acceptable for publication in EMBO Molecular Medicine.

Please note that given the extensive work required for the revisions and her previous participation, Dr. Monika Kuciak now also shares first authorship. The initial key contributions of Dr. Christophe Mas are also acknowledged by inclusion of the mention of the patient application that the University of Geneva has put forward.

We thank you for the work on our submission and for the decision to move forward. We agree that while Referee 1 comments on important issues in the long run, these are for future experimentation and analysis. We thus proceed to reply to referees 2 and 3 below.

Referee #1:

The authors have performed a drug repositioning screen to attempt to identify compounds that might interfere with the WNT-beta catenin pathway. They have identified ivermectin and related compounds as potent inhibitors of signalling downstream of WNT. They have tested with appropriate controls both in vitro and in vivo the activity of the compounds and reasonably conclude that the compounds effects are specific. Unfortunately at this stage the manuscript fails to address a number of essential points and therefore in my opinion is not suitable for publication. The authors might want to consider the following: a. pursue the identification of the molecular target; b. perform detailed pharmacokinetics and pharmacodynamic studies, e.g. Cyclin D1 staining in tissue sections, to also address issues of drug penetration vs. impact on tumour initiation vs. maintenance; c. understand impact on cancer stem cells if any, by performing required customary experiments , e.g. TIC frequency, etc. most importantly, attempt to understand lack of efficacy in vivo, as pursuing these compounds as potential therapeutics needs this question to be solved now rather than later. The manuscript would also benefit from a thorough revision of English style and grammar.

We thank this referee for raising important issues for future analysis. The paper has been revised and the language improved where possible. We note Referee 3 found the paper 'well written'.

Referee #2:

The work by Melotti, Mas et al. describes a repositioning screening strategy which identifies a class of macrocyclic compounds that can potently inhibit the Wnt signalling pathway. Overall this is an excellent study that has led to the identification of a clinically-experienced, off-patent compound with inhibitory activity. This study will provide a platform for identification of the specific molecular target of Ivermectin and allow further development of more efficacious Wnt signalling pathway inhibitors. I have only minor points to be addressed as follows:

We thank this referee for his/her support.

1) Beta-CATENIN is not hyphenated in the intro, but everywhere else. Page 5, pole should read pool (2 instances).

These typos have been corrected. Thank you for pointing this out.

However, we believe pole is correctly used in the sentence below on p.5: 'i- a reliable dynamic range determined by activated b-CATENIN as the activating **pole** and activated b-CATENIN plus dominant-negative TCF (dnTCF) as the repressing **pole**, and ii- sine qua non mimicry of genetic blockade by dominant-negative TCF (dnTCF) activity for any hit.'

2) Was Ivermectin in the screening library? If so, how did it score in the luciferase reporter assay?

No, it was not included in the tested collection of compounds.

3) Structure-function analyses make use of proliferation assays (BrdU incorporation) and are not complete with respect to specific Wnt pathway activity. Could the authors include luciferase reporter assay data for Ivermectin and Moxidectin, Bryostatin, Doramecting to allow direct comparison alongside proliferation assays? I realize this is largely covered with target gene analysis however, for direct efficacy comparison between compounds, the relative expression of individual target genes does not appear to be consistent.

This is an important point that we have addressed by analysing the expression of three TCF target genes in response drug action. We believe this is a much more direct and relevant measure for the activity and effects of the different drugs rather than indirect luciferase assays utilizing an artificial multimerized TCF binding site. Moreover, given that primary cells are very difficult to transfect with TOP/FOP constructs (or any constructs), we have opted to test the endogenous expression of *AXIN2, LGR5* and *p21* in two cell types, primary CC14 and cell line Ls174T. The results are now shown in the new Fig. 2 and described in the text. Ivemerctin, Moxidectin and Doramectin have similar activities whereas Bryostatin is inactive, further supporting our original data.

4) Are the panels for 3 C, E needed - ie. are there visual differences between the treatments?

We have removed the second set of spheroid images from Fig. 3.

5) Ls174T cells harbour the S45 mutation in beta-catenin. Do the primary cells (CC14) which are insensitive to Ivermectin in xenograft assays also harbour mutations in CTNNB1 or are they APC-mutant, similar to the Ivermectin-sensitive DLD1 (and HT-29) cell lines?

We have sequenced the relevant areas of these genes and have found no mutations in either *APC* or *b-CATENIN.* This is now mentioned in the text and described in Materials and Methods.

6) Is Wnt pathway activity sensitive to Ivermectin with overexpression of the LEF DNA binding domain fused to the C-terminus of beta-CATENIN (such as in [Hao et al (2013) Cell Reports])?

We thank this referee for pointing out this interesting avenue of research. We believe that a careful structure-function analysis requires detailed experimentation out of the scope of this paper.

7) The effects of okadaic acid, forskolin and potentially Ivermectin treatment are likely pleitropic and the data implicating the effect of Ivermectin mediated by the action of a phosphatase on C-terminal phosphorylation sites is largely correlative. Moreover, it is not clear from the literature whether the effect of C-terminal phosphorylation affects cellular levels, nuclear localization, the interactions or transcriptional activity of beta-CATENIN. What would be the effect of Ivermectin on expressed versions of beta-CATENIN (or the construct in point 6)) with mutant C-terminal phosphorylation sites (ie. converted to Ala versus Asp)?

This referee suggests interesting additional studies that should be the focus of future studies. Indeed, exactly what does Ivermectin bind in vivo remains to be determined, as explicitly stated in the main text.

Referee #3:

The authors identify and validate commonly used anti-helminthic compounds as novel inhibitors of TCF-beta-catenin activity. The study is highly significant as the Wnt pathway has been implicated in a wide variety of human cancers but few approaches to target this pathway have been developed. Furthermore, many of the compounds are currently under investigation target Wnt signalling at the cell-surface and will likely not be effective in tumour cells that carry mutations or activating events that lie downstream of Fzd receptors. Moreover, the authors' findings that ivermectin and selamectin can inhibit the transcriptional activity of beta-catenin/TCF are highly clinically relevant as these agents are already in clinical use. Overall, the studies are well done and the manuscript is well written.

Although the precise mechanism by which these agents inhibit Wnt signalling is not precisely defined in this manuscript, the authors do a good job demonstrating that the effects of these agents are specific. Furthermore, the main point of the submission, that these common drugs inhibit the Wnt pathway, is significant and well worth reporting.

We thank this referee for her/his support.

Minor comments include:

1. The compounds were screened based on the inhibition of proliferation. Given their effects on clonogenic tumour growth in vitro and in vivo, it would be useful to know if either of these compounds also induces apoptosis at the doses that inhibit Wnt target gene expression.

As indicated by this referee, we have performed analyses of activated Caspase3 as a measure of apoptosis in response to drug action. Ivermectin and Selamectin induce increases in the number of apoptotic cells. This is now included in the text in a new section and shown in the new Fig. 2.

2. For the studies examining clonogenic growth, examination of serial sphere formation would be useful in defining the inhibition of self-renewal by beta-catenin blockade.

As suggested, we have performed secondary clonogenic assays in Ls174T cells and show that the number of secondary clones, unlike the number of primary clones, is not affected by pre-treatment with Ivermectin. This result indicates the absence of long-term effects of the drugs beyond the reduction in the number of clonogenic cancer stem cells when the drugs are present. This data is shown in the new Fig. 3.

3. Similarly, an examination of potential colon cancer CSC marker, such as CD133, may bolster the claims that these cells are undergoing inhibition.

We have performed quantification of $CD133^+$ cells by MACS in two different cell types (Ls174T and CC14) in triplicate using two different methods to dissociate the cells (Trypsin and Acutase). In all cases, we find no difference in the percentages of $CD133^+$ cells in control vs Ivermectin treated cells. This is now mentioned in the text, along discussion of this result, which separates $CD133^+$ expression from spheroid-forming capacity.

4. In terms of Wnt signalling, was localization of beta-catenin impacted in any way by the compounds?

Following this referee's suggestion, we have performed immunolocalization of b-CATENIN in control CC14 and Ls174T cells and in those treated with Ivermectin. There are no clear differences in the distribution patterns of this protein between the two samples. This result is shown in the new Fig. 6.

2nd Editorial Decision 08 July 2014

Thank you for the submission of your manuscript to EMBO Molecular Medicine. We have now heard back from the two Reviewers whom we asked to evaluate your manuscript.

As you will see the Reviewers are now satisfied with your manuscript and I am thus prepared to accept your manuscript for publication pending the following amendments:

1) There is excessive contrasting in Western blots in Fig.s 4, 5 and 6. Please provide better figure images.
2) The x axis legend (numbers) in Fig 5D is blocky when zooming in. Please provide a better figure image
3) The control lanes in Fig. S8B appear to have been juxtaposed ex post to the treated lanes. Please make that sure that these discontinuities are better demarcated (e.g. with a vertical black line) and explained in the relative legend. Also, please provide the source data for this figure.
4) Every published paper now includes a 'Synopsis' to further enhance discoverability. Synopses are displayed on the journal webpage and are freely accessible to all readers. They include a short standfirst - to be written by the editor - as well as 2-5 one sentence bullet points that summarise the paper (to be written by the author). Please provide the short list of bullet points that summarise the key NEW findings. The bullet points should be designed to be complementary to the abstract - i.e. not repeat the same text. We encourage inclusion of key acronyms and quantitative information. Please use the passive voice. Please attach these in a separate file or send them by email, we will incorporate them accordingly.
5) We are now encouraging the publication of source data, particularly for electrophoretic gels and blots, with the aim of making primary data more accessible and transparent to the reader. Would you be willing to provide a PDF file per figure that contains the original, uncropped and unprocessed scans of all or at least the key gels used in the manuscript? The PDF files should be labeled with the appropriate figure/panel number, and should have molecular weight markers; further annotation may be useful but is not essential. The PDF files will be published online with the article as supplementary "Source Data" files. If you have any questions regarding this just contact me.

Please submit your revised manuscript within two weeks. I look forward to seeing a revised form of your manuscript as soon as possible.

***** Reviewer's comments *****

Referee #2 (Remarks):

The rebuttal to my review is fine, I would have liked to see a bit more mechanistic detail (such as points (6) and (7) of my original review), however I accept the authors' explanation that these may be incidental to this study. One editing problem - on the PDF I have there are font problems with most of the text in Fig. 2.
Having read Reviewer 1's suggestions I agree that these would increase the impact and scope of this study, however accommodation of these experiments into the manuscript would require removing key data from the original 6 Figures. It is my opinion that in its current state the study as a whole is complete- the authors have identified Ivermectin in a drug screen and validated it as an inhibitor of Wnt-dependant signalling/proliferation/apoptosis/clonal growth, in vivo and in vitro. Target identification and pharmacological studies as well as further validation of TICs as targets of the drug will provide future avenues of research.

Referee #3 (Remarks):

In this revised manuscript, I believe that the issues raised during the previous review have been more than adequately addressed. I believe that the manuscript should be accepted.

2nd Revision - authors' response 14 July 2014

Thank you for the comments of the referees and for your acceptance of our paper.

Attached you will find revised files as requested, including the source data for the panel mentioned in your letter. The new figure is included in the Supplementary Material file.

Western blot panels in Figs 4,5,6 have been redone with as little contrast as possible. GAPDH is highly abundant and the signal is very clean with very short exposures.

The axes of Fig5D have been improved as requested.

The text in Fig2 has been also updated.

Al figures revised for accuracy and detail. In revising them I discovered a small error in Fig. S6B that I have corrected. The right panel had been duplicated by mistake from Fig 5.

The main text has been revised.

We trust our paper is now ready for publication.
